# Cooperation between Paxillin-like Protein Pxl1 and Glucan Synthase Bgs1 Is Essential for Actomyosin Ring Stability and Septum Formation in Fission Yeast

**DOI:** 10.1371/journal.pgen.1005358

**Published:** 2015-07-01

**Authors:** Juan C. G. Cortés, Nuria Pujol, Mamiko Sato, Mario Pinar, Mariona Ramos, Belén Moreno, Masako Osumi, Juan Carlos Ribas, Pilar Pérez

**Affiliations:** 1 Instituto de Biología Funcional y Genómica, Consejo Superior de Investigaciones Científicas (CSIC) / Universidad de Salamanca, Salamanca, Spain; 2 Department of Ciències Mèdiques, Bàsiques,Institut de Recerca Biomèdica (IRB) de Lleida, Faculty of Medicine, University of Lleida, Lleida, Spain; 3 Laboratory of Electron Microscopy/Open Research Centre, and Department of Chemical and Biological Sciences, Japan Women’s University, Mejirodai, Bunkyo-ku, Tokyo, Japan; 4 Centro de Investigaciones Biológicas, Consejo Superior de Investigaciones Científicas (CSIC), Madrid, Spain; Howard Hughes Medical Institute and Vanderbilt University School of Medicine, UNITED STATES

## Abstract

In fungal cells cytokinesis requires coordinated closure of a contractile actomyosin ring (CAR) and synthesis of a special cell wall structure known as the division septum. Many CAR proteins have been identified and characterized, but how these molecules interact with the septum synthesis enzymes to form the septum remains unclear. Our genetic study using fission yeast shows that cooperation between the paxillin homolog Pxl1, required for ring integrity, and Bgs1, the enzyme responsible for linear β(1,3)glucan synthesis and primary septum formation, is required for stable anchorage of the CAR to the plasma membrane before septation onset, and for cleavage furrow formation. Thus, lack of Pxl1 in combination with Bgs1 depletion, causes failure of ring contraction and lateral cell wall overgrowth towards the cell lumen without septum formation. We also describe here that Pxl1 concentration at the CAR increases during cytokinesis and that this increase depends on the SH3 domain of the F-BAR protein Cdc15. In consequence, Bgs1 depletion in cells carrying a *cdc15_ΔSH3_* allele causes ring disassembly and septation blockage, as it does in cells lacking Pxl1. On the other hand, the absence of Pxl1 is lethal when Cdc15 function is affected, generating a large sliding of the CAR with deposition of septum wall material along the cell cortex, and suggesting additional functions for both Pxl1 and Cdc15 proteins. In conclusion, our findings indicate that CAR anchorage to the plasma membrane through Cdc15 and Pxl1, and concomitant Bgs1 activity, are necessary for CAR maintenance and septum formation in fission yeast.

## Author Summary

Cytokinesis requires assembly of an actomyosin ring adjacent to the plasma membrane, which upon contraction pulls the membrane to form a cleavage furrow. In fungi ring closure is coordinated with the synthesis of a cell wall septum. Knowledge about the molecules anchoring the ring to the membrane is very limited. We have found that fission yeast paxillin, located at the ring, and Bgs1, the enzyme responsible for primary septum formation, located at the membrane, cooperate during cytokinesis. Both are required to anchor the ring to the membrane and to maintain it during cytokinesis. Moreover, both proteins cooperate to form the septum. Accordingly, paxillin is essential when Bgs1 is depleted. When both proteins are missing, the contractile ring forms but the lateral cell wall overgrows inwards without a defined cleavage furrow and septum formation. During cytokinesis there is an increase of paxillin which depends on the SH3 domain of the F-BAR protein Cdc15. Consequently the absence of this domain mimics the phenotype of paxillin absence in Bgs1-depleted cells. Interestingly, a decreased function of both Cdc15 and paxillin uncouples the septum synthesis from the ring contraction, indicating an essential cooperation between these proteins and Bgs1 for proper cytokinesis.

## Introduction

Cytokinesis is the final stage of the eukaryotic cell cycle, when a mother cell separates into two daughter cells. Cytokinesis is mediated by a contractile actomyosin ring (CAR) that is conserved between fungal and animal cells [1]. In addition to CAR contraction, fungal cells assemble a division septum wall which is essential for cell integrity [2]. Recent work proposed that the pulling force from CAR contraction is not sufficient to accomplish cytokinesis and that a pushing force is also necessary [3], and we showed that support of the lateral cell wall is crucial for proper cytokinesis [4].

Fission yeast CAR is composed of many proteins besides F-actin and heavy and light chains of myosin II [5,6]. Significant progress in identifying and characterizing the proteins that participate in CAR positioning, assembly, stabilization, and integrity has already been made [1,7,8]. The septum of fission yeast is a three-layered polysaccharide structure made of a middle primary septum (PS) flanked by two secondary septa (SS), one on each side. Both the PS and SS are formed by essential β-glucans. The enzyme involved in their formation is the β(1,3)glucan synthase, composed of at least a regulatory and a catalytic subunit. The former is the GTPase Rho1 [9,10]. Fission yeast contains four different catalytic subunits named Bgs1 to Bgs4. Bgs1 is responsible for the linear β(1,3)glucan necessary for PS formation [11]; Bgs4 builds branched β(1,3)glucan [12], which is the most abundant polymer in the septum and cell wall. During cytokinesis this polymer is required for connecting the CAR to the extracellular cell wall, for SS formation, for the correct PS structure, and to maintain the cell integrity during cell separation [4]. Ags1 synthesizes α-glucan, which is also a major cell wall polymer [13,14]. During cytokinesis α-glucan is essential for the PS adhesion strength needed to withstand the internal turgor pressure during cell abscission, for the SS structure, and for cell integrity [15].

While much is known about the protein components of the CAR, how these proteins coordinate and interact with the septum synthesis enzymes to form the cleavage furrow remains unclear. A key protein for CAR positioning and function is the F-BAR domain-containing phosphoprotein Cdc15 [16]. Cdc15 is a membrane-anchored scaffold for CAR assembly and links the ring to the plasma membrane through its essential F-BAR domain [17]. Dephosphorylation of Cdc15 at mitotic entry, mediated by Clp1, induces a conformational switch in the protein that promotes oligomerization [18]. Cdc15 also participates in endocytosis [19], and it has recently been shown that it localizes to the Golgi where it helps in the delivery of Bgs1 to the plasma membrane during cytokinesis [20]. Additionally, Cdc15 collaborates with the F-BAR domain-containing Imp2 [21] in CAR attachment to the membrane through their C-terminal SH3 domains, which mediate interactions with the paxillin homolog, Pxl1, and the C2-domain-containing protein Fic1 [22]. Another F-BAR domain protein, Rga7, associates with Imp2 in a protein complex that coordinates the late stages of cytokinesis [23].

Animal cell paxillin is a multi-domain scaffold protein that localizes to the intracellular surface of the plasma membrane in sites of cell adhesion of extracellular matrix [24]. Fission yeast Pxl1 is a component of the CAR that shares with other paxillins the Lin-11, Isl-1, Mec-3 (LIM) domains in its C-terminal half but presents a different N-terminal region, which is required for binding to the Cdc15 and Imp2 SH3 domains [22]. Pxl1 also binds to the type II myosin Myo2 and is necessary for CAR integrity and proper constriction [25,26].

In this work, we show that the functions of Pxl1 in the CAR and Bgs1 in the membrane are necessary for stable CAR anchorage. Additionally, cooperation between Pxl1 and Bgs1 is shown to be essential for CAR maintenance and septum formation, since cells without Bgs1 display CAR disassembly and cannot form septa when Pxl1 is absent or when the Pxl1 levels in the ring are reduced, as in the case of cells carrying the defective *cdc15*_*ΔSH3*_ allele.

## Results

### Pxl1 Is Required for Stable CAR and Septum Positioning in the Middle of the Cell

**Fig 1:**
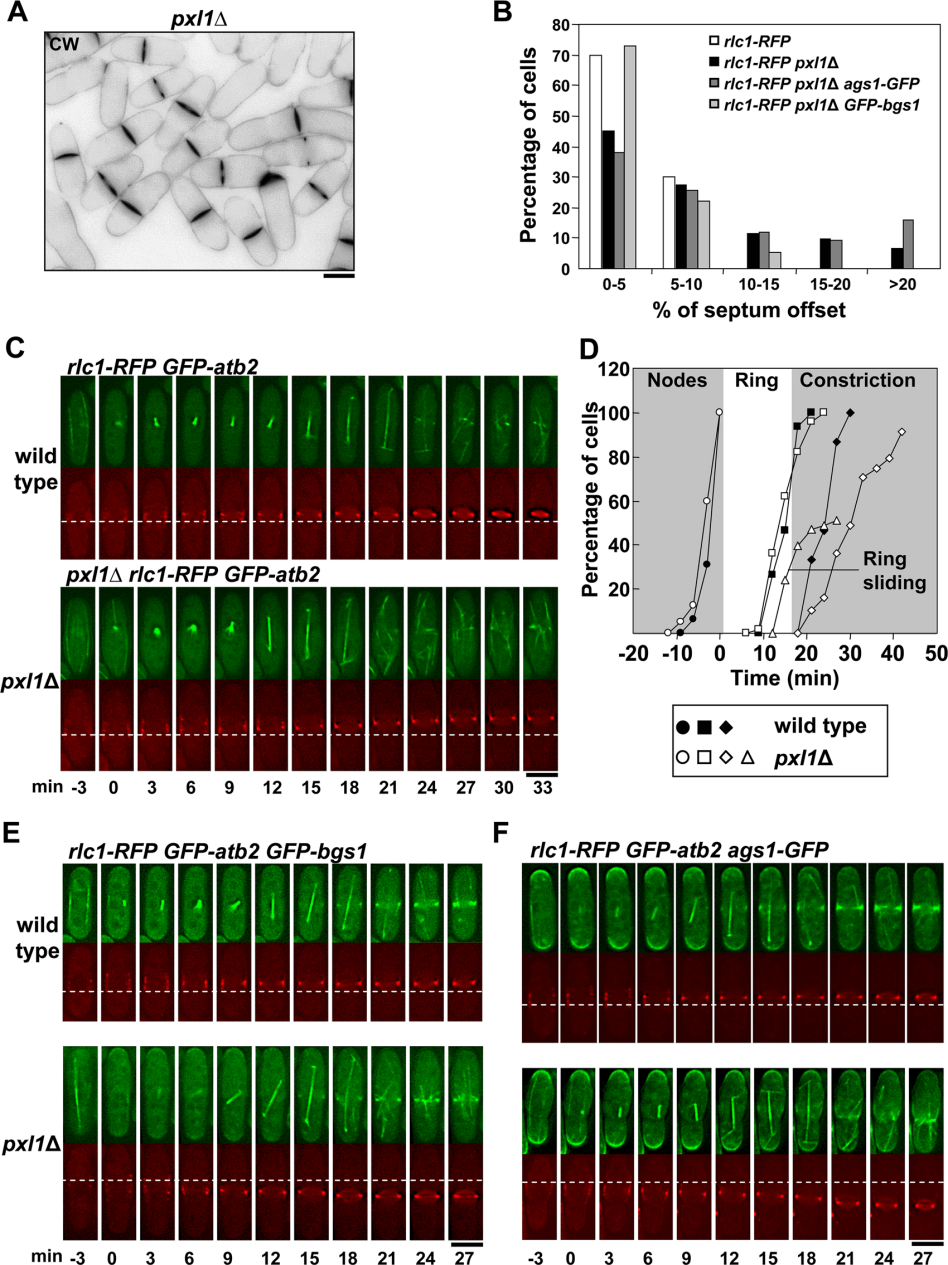
Pxl1 is required for stable CAR and septum positioning in the middle of the cell. **(A)** A Calcofluor White (CW) staining image of *pxl1*Δ cells with off-centered septa. **(B)** Histogram showing the indicated intervals of septum position measured as the percent of septum offset from the cell center: white bars, wild-type cells (n = 40); black bars, *pxl1*Δ cells (n = 142); dark grey bars, *pxl1*Δ *ags1* ^+^-*GFP* cells (n = 131); and light grey bars, *pxl1*Δ *GFP-bgs1* ^+^ cells (n = 59). The position of the septum was measured with Image J software as described in the Materials and Methods section. **(C)** Time series of fluorescence micrographs (one medial z slide, 3 min intervals) of cells carrying Rlc1-RFP and GFP-Atb2. The first panel shows a wild-type cell with a centrally located ring that began to constrict at +24 min. The second panel shows a *pxl1*Δ cell with a ring that moved toward the upper pole at +15 min. Spindle microtubules appear at time 0. **(D)** Time courses of appearance of cortical nodes tracked with Rlc1-RFP (circle), completion of ring (square), onset of ring constriction (diamond), and ring sliding (triangle). Filled symbols are wild-type cells (circle n = 16; square n = 15; diamond n = 15), and open symbols are *pxl1*Δ cells (circle n = 40; square n = 50; diamond n = 45; triangle n = 27). **(E)** Time series of fluorescence micrographs (one medial z slide, 3 min intervals) of cells carrying GFP-Bgs1, Rlc1-RFP and GFP-Atb2. The first panel shows a wild-type cell where Bgs1 was detected in the cell middle at +15 min. The second panel shows a *pxl1*Δ cell with a ring that moved toward the lower pole at +18 min and where Bgs1 was detected in the cell middle at +15 min. Spindle microtubules appear at time 0. **(F)** Time series of fluorescence micrographs (one medial z slide, 3 min intervals) of cells carrying Ags1-GFP, Rlc1-RFP and GFP-Atb2. The first panel shows a wild-type cell where Ags1 was detected in the cell middle at +12 min. The second panel shows a *pxl1*Δ cell with a ring that moved toward the lower pole at +18 min where Ags1 was detected at +15 min. Spindle microtubules appear at time 0. Dashed line: reference for the ring position. Elapsed time is shown in minutes. Scale bars, 5 μm.

*Schizosaccharomyces pombe* paxillin, Pxl1, is a Rho1 negative regulator that contributes to the maintenance of CAR integrity [25,26]. Cells lacking Pxl1 present a higher proportion of septated cells (45 to 50%) and the CAR constriction rate is slower than in wild type cells. These phenotypes are likely due to a defective actomyosin ring that occasionally splits into two [25,26]. A detailed observation of cells lacking Pxl1 labeled with Calcofluor White (CW) so the primary septum (PS) could be seen, revealed some cells with off-center septa (Fig 1A). While all wild type cells formed septa within 10% offset from the cell center, only 70% of the cells lacking Pxl1 formed septa in this section of the cell (Fig 1B). This phenotype was further analyzed by following CAR assembly in time-lapse experiments. Wild type and *pxl1*Δ cells carrying GFP-Atb2 (tubulin) and Rlc1-RFP (CAR) were adjusted to a timescale where zero corresponds to the spindle formation and spindle pole body separation. CARs from *pxl1*Δ cells assembled from condensed nodes as they do in wild type cells. However, once formed, *pxl1*Δ CARs delayed contraction and moved slightly along the longitudinal axis, suggesting that ring anchorage to the membrane was not steady (Fig 1C and 1D, dashed line). CAR sliding occurred until the septum was visible with CW in *pxl1*Δ cells (S1A Fig). No CAR sliding was observed in wild type cells (Fig 1C and 1D).

It has been described that glucan synthase Bgs1 participates in CAR stability [20], therefore we analyzed the localization of this synthase and the α-glucan synthase Ags1, which is also required for a correct PS formation [15], in *pxl1*Δ cells. Both synthases are observed in the division area before the PS can be detected [15,27]. Time-lapse microscopy of *pxl1*Δ cells carrying Rlc1-RFP, GFP-Atb2, and GFP-Bgs1 during cytokinesis showed that GFP-Bgs1 moved with the CAR (Fig 1E). Interestingly, CAR sliding was not as pronounced in *pxl1*Δ with GFP-tagged Bgs1 as it was in cells with untagged Bgs1 (Fig 1B). Moreover, GFP-tagged Bgs1 partially suppressed *pxl1*Δ septation phenotypes (S1B Fig). Perhaps the GFP tag improves Bgs1 stability and/or function. In agreement, it has been described that GFP-Bgs1 also partially rescues the morphological defects of *rng2ΔIQ* mutant cells defective in cytokinesis, and partially corrects its slow rate of ring constriction [28].

Ags1-GFP also accompanied the CAR movement in *pxl1*Δ cells and slid along the membrane until PS was detected (Fig 1F and S1A Fig, arrowhead). Therefore, in the absence of Pxl1 the CAR was not stably positioned and slid along the membrane together with the glucan synthase enzymes until the onset of septation.

### Correct Bgs1 Function Is Important for CAR Integrity and Maintenance of Ags1 and Bgs4 in the Septum Membrane

**Fig 2:**
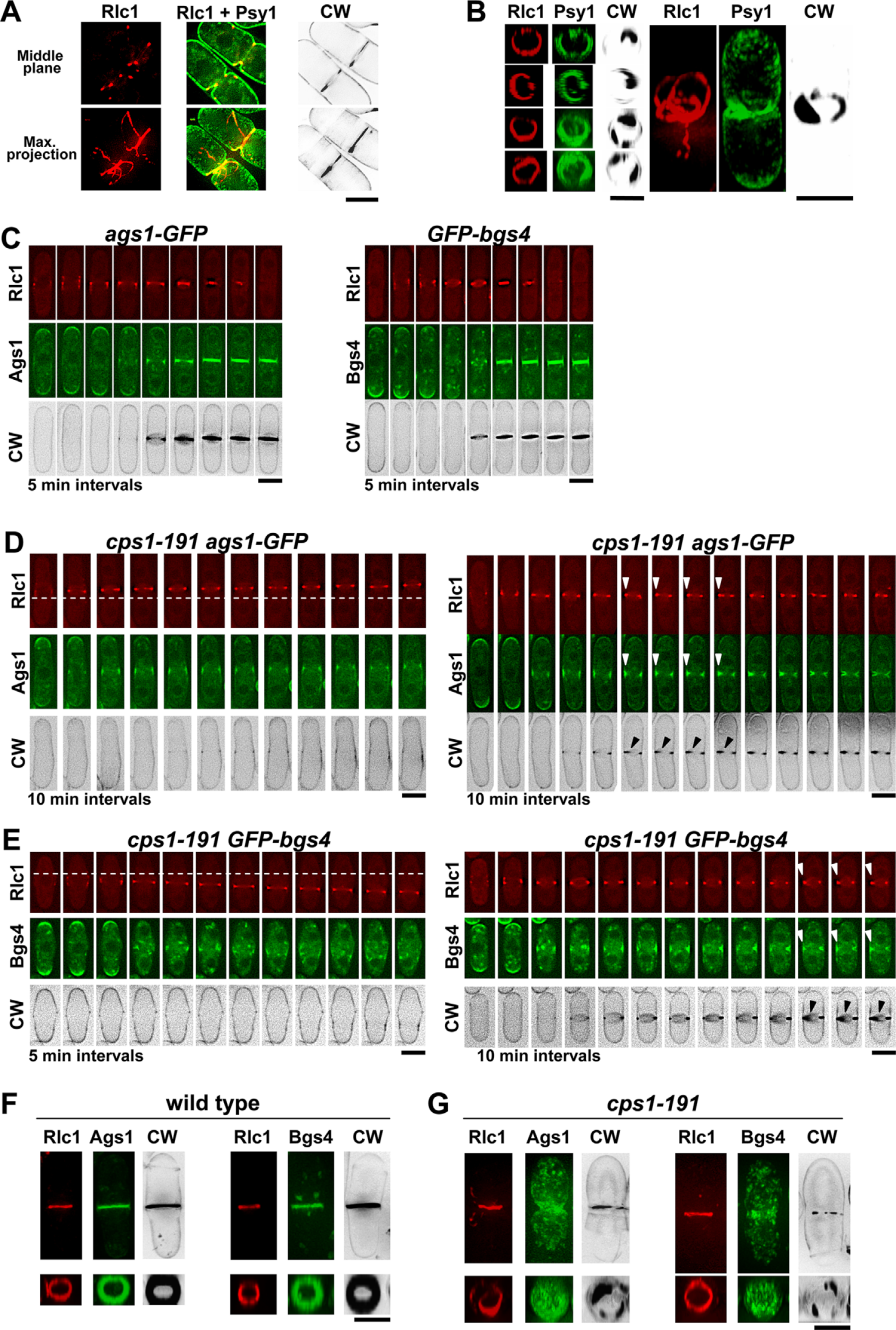
Correct Bgs1 function is important for CAR Integrity and maintenance of Ags1 and Bgs4 in the septum membrane. **(A)** Fluorescence micrographs of CW stained *cps1-191* cells carrying Rlc1-RFP (ring) and GFP-Psy1 (plasma membrane). *cps1-191* cells growing in YES+S (S = 1.3M sorbitol) at 25°C were shifted to 37°C for 6 h and imaged. Medial z slide (upper panels) and maximum-intensity projections of 28 z slides at 0.3 μm intervals (lower panels) of the same cells are shown. **(B)** Three-dimensional reconstructions (28 z slides at 0.3 μm intervals) of cells grown as in A. Left, equatorial plane; right, longitudinal plane. **(C-E)** Time-lapses (one medial z slide, 5 min intervals, although in some cases only is shown 10 min interval as indicated) of CW-stained wild-type **(C)** and *cps1-191* (**D** and **E**) cells carrying Rlc1-RFP and Ags1-GFP or GFP-Bgs4. Wild type and *cps1-191* cells growing in YES+S at 25°C were shifted to 37°C for 1.5 h and filmed to capture ring formation, and Ags1 or Bgs4 arrival to the division site. Two *cps1-191* cells showing a sliding ring which does not constrict (left in D and E) and two *cps1-191* cells with a slow ring constriction and septum deposition (right in D and E) are shown. Arrowheads indicate synthesis of the septum wall in the absence of ring constriction. A dashed line is drawn as reference for the ring position. **(F** and **G)** Fluorescence micrographs of CW-stained wild-type (F) and *cps1-191* (G) cells carrying Rlc1-RFP, and GFP-Bgs4 or Ags1-GFP. Cells were grown as in A. Maximum-intensity projections of 28 z slides at 0.3 μm intervals (upper panels) and the corresponding three-dimensional reconstructions of the equatorial plane including the septum area (lower panels) of the complete cell are shown. In all the three-dimensional reconstructions, from the 28 slides acquired it was only selected those slides that cover the entire cell diameter. Scale bars, 5 μm.

Previous work identified a synthetic lethal interaction between *pxl1*Δ and different *bgs1/cps1* thermosensitive mutants [26], suggesting that Pxl1 and Bgs1 collaborate in an essential process. To test this hypothesis, we first analyzed the septation defects of cells carrying the *bgs1* thermosensitive allele *cps1-191*. These cells were grown at the restrictive temperature (37°C) in the presence of 1.3M sorbitol (S) to allow the deposition of the septum (Fig 2A and 2B). In cells carrying Rlc1-RFP and GFP-Psy1 (a syntaxin homolog that is a marker for the plasma membrane [29]) the processes of membrane invagination and septum formation were accompanied by a disorganized ring which appeared fragmented, with Rlc1 strands connected to the ring. These CAR defects were similar to those of *pxl1*Δ cells [25,26]. Therefore, a defective Bgs1 function affects the CAR position in the cell middle as was already described [20], and also affects the ring integrity during septum ingression. Because other glucan synthases, such as Ags1 and Bgs4, must collaborate in the formation of the aberrant septum structures observed in *cps1-191* cells, we analyzed the localization of these synthases, before and during septation, in wild type and *cps1-191* cells carrying Rlc1-RFP grown at the restrictive temperature for 1.5 h (Fig 2C, 2D, and 2E). At this time point, *cps1-191* cells made new CARs that were maintained for a long time. Some cells formed rudimentary or partial depositions of CW-stained septa without a proper CAR constriction (Fig 2D and 2E, arrowheads). Time-lapse images of *cps1-191 rlc1* ^+^ *-RFP* cells carrying Ags1-GFP or GFP-Bgs4 showed that both synthases localized to the division area as in wild type cells (Fig 2C, 2D, and 2E). Ags1 concentrated and moved with the ring along the plasma membrane, and in cells with septum deposition it remained concentrated at the septum area (Fig 2D). In agreement with previous observations [12], Bgs4 did not concentrate in the cell membrane of cells without septum deposition (Fig 2E, left) but did appear concentrated in cells with a partial septum (Fig 2E, right). After longer times (6 h) at the restrictive temperature, both synthases spread along the septum membrane and were also detected in cytoplasmic vesicles. They did not form a homogeneously filled disk-like surface as they do in wild type cells, but an irregularly filled disk indistinguishable from the numerous cytoplasmic vesicles located in the middle (Fig 2F and 2G). Therefore, Bgs1 function is required for ring integrity, for the correct synthesis of the PS, and for the proper localization of Ags1 and Bgs4 as a homogeneous disk structure in the membrane during septum ingression.

### Cooperation of Bgs1 and Pxl1 Is Essential for CAR Maintenance and Septum Formation

**Fig 3:**
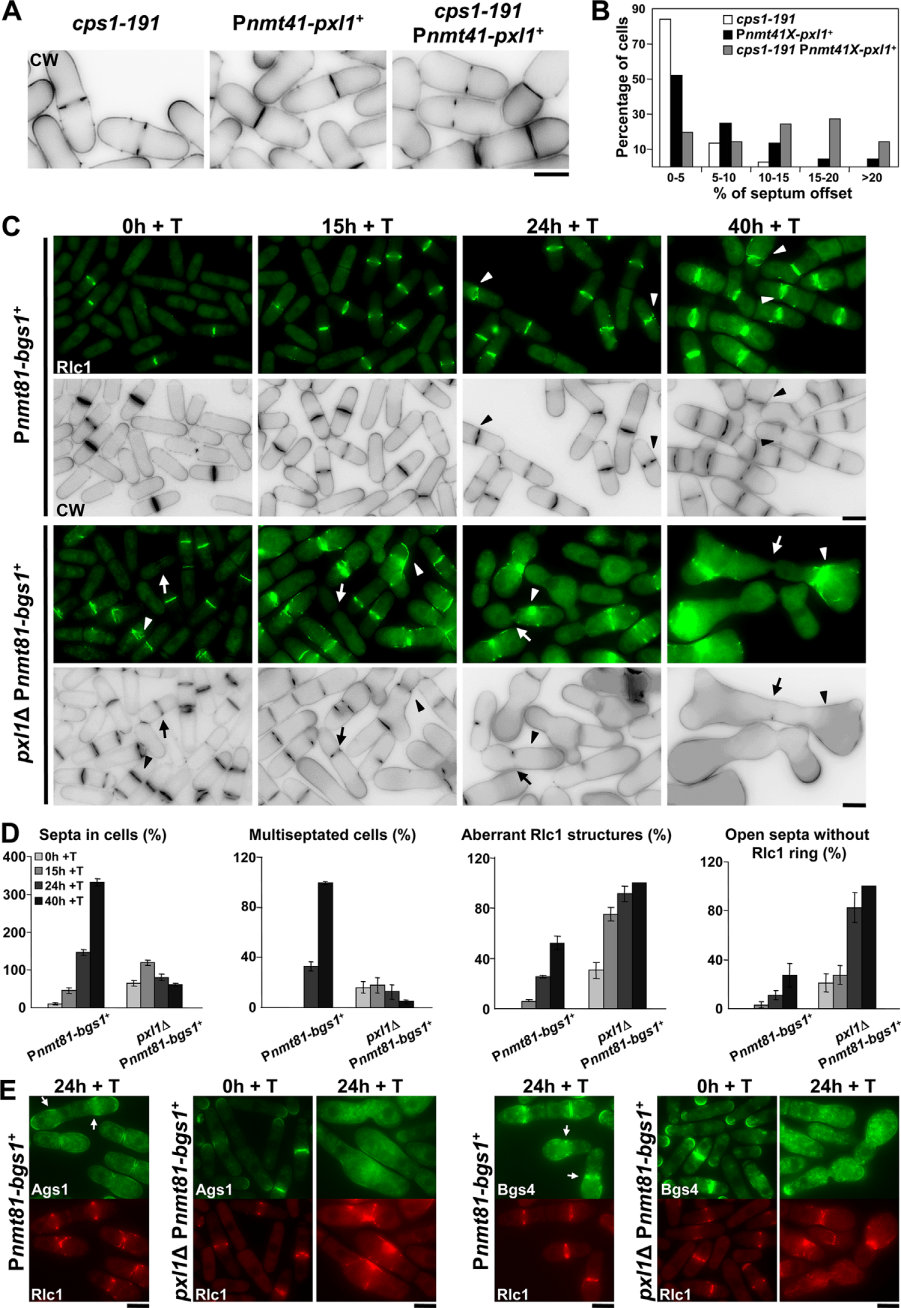
Cooperation of Bgs1 and Pxl1 is essential for CAR maintenance and septum formation. **(A)** CW staining images of *cps1-191*, P*nmt41-pxl1* ^+^ and *cps1-191* P*nmt41-pxl1* ^+^ cells. Cells were grown at 25°C (permissive temperature for *cps1-191*) in the presence of thiamine (+T, *pxl1* ^+^ repressed) for 24 h and imaged. **(B)** Histogram showing the indicated intervals of positions of the septa measured as the percent of septum offset from the cell center: white bars, *cps1-191* cells (n = 37); black bars, P*nmt41-pxl1* ^+^ cells (n = 44 cells); and grey bars, *cps1-191* P*nmt41-pxl1* ^+^ cells (n = 62 cells). The percentages of septum offset were calculated as described for the Fig 1B. **(C)** Fluorescence micrographs of P*nmt81-bgs1* ^+^ and *pxl1*Δ P*nmt81-bgs1* ^+^ cells stained with CW and carrying Rlc1-GFP. Cells were grown to early log-phase in EMM+S (time 0 h), shifted to EMM+S+T for *bgs1* ^+^ repression (times 15, 24 and 40 h + T), and imaged at the indicated times. Arrow: Cell with an open septum without the ring of Rlc1. Arrowhead: Cell with an aberrant ring of Rlc1. **(D)** Histograms showing the indicated percentages of septa and Rlc1 structures in P*nmt81-bgs1* ^+^ (n = 150 cells or hypha units for each time), and *pxl1*Δ P*nmt81-bgs1* ^+^ cells (n = 100 cells or hypha units for each time). Note that P*nmt81-bgs1* ^+^ strains at 24 h of *bgs1* ^+^ repression appear as hypha units, each being equivalent to several single cells. (**E**) Fluorescence micrographs of *Pnmt81-bgs1* ^+^ (time 24 h of *bgs1* ^+^ repression) and *pxl1*Δ *Pnmt81-bgs1* ^+^ (times 0 and 24 h of *bgs1* ^+^ repression) cells carrying Rlc1-RFP and Ags1-GFP or GFP-Bgs4. Cells were grown as in C and imaged at the indicated times. Arrow: concentration of Ags1-GFP or GFP-Bgs4 in the septum membrane. Scale bars, 5 μm.

To study the functional interaction of Bgs1 and Pxl1, we generated a strain where the *pxl1* ^+^ open reading frame was expressed under the control of the *nmt1-41* promoter [30]. Repression of this promoter by adding thiamine showed septation defects close to those of *pxl1*Δ cells with around 20% off-center septa (Fig 3A and 3B). However, when *pxl1* ^+^ repression was performed in a *cps1-191* background at the permissive temperature for the *cps1-191* mutation (25°C), the percentage of off-center septa increased dramatically, with septa displaced further than 10% from the middle in more than 60% of the cells (Fig 3A and 3B). Therefore, a minimal alteration of Bgs1 function aggravates the defects caused by *pxl1* ^+^ repression, suggesting that both proteins collaborate in maintaining the CAR position in the cell middle.

We also studied the role of Pxl1 and Bgs1 during septation by decreasing the level of Bgs1 protein in cells lacking Pxl1. A strain where the *bgs1* ^+^ gene was expressed under the *nmt1-81* promoter was used [31]. Repression of this promoter generates a more than 300-fold Bgs1 reduction up to undetectable levels after 15 h in the presence of thiamine but cells remain viable for a long period (over 60 h) [11]. P*nmt81-bgs1* ^+^ repression was examined in both wild type and *pxl1*Δ cells carrying Rlc1-GFP and stained with CW. As described previously, septum completion was observed in cells during all stages of *bgs1* ^+^ repression, although the structure of those septa was different from that of normal septa ([11], and Fig 3C). After 15 h of *bgs1* ^+^ repression most cells contained one septum. At longer times (40 h + T) the cells contained multiple septa and branched, generating hyphae (Fig 3C). At all the times during *bgs1* ^+^ repression, GFP-Pxl1 was observed forming a correct ring, which suggests that its localization is independent of Bgs1 (S2 Fig). However *bgs1* ^+^ repression in *pxl1*Δ cells caused a significant decrease in the number of septa (Fig 3C and 3D). Interestingly, after 15 h with thiamine many *pxl1*Δ P*nmt81-bgs1* ^+^ cells displayed open septa with an aberrant Rlc1 structure, or without a CAR (Fig 3C, arrowheads and arrows). These defects were more pronounced after 24 h with thiamine, when we observed some cells that had aberrant Rlc1 structures without centripetal synthesis of the PS and most cells presented open septa without a CAR (Fig 3C and 3D). At longer times (40 h) with thiamine *pxl1*Δ P*nmt81-bgs1* ^+^ cells elongated and swelled but did not form septa, and Rlc1 appeared mostly as aberrant accumulations (Fig 3C and 3D). These results indicate that Bgs1 and Pxl1 cooperate to maintain the position and integrity of the CAR during cytokinesis, and more important, they are together essential for the synthesis of the entire septum structure.

In *Saccharomyces cerevisiae* a temporary inactivation of Rho1 GTPase is required during septum ingression [32]. Because deletion of Pxl1 increases Rho1 activity [26], this could be impeding septum ingression upon *bgs1* ^+^ repression in *pxl1*Δ cells. However, deletion of the Rho-GAP Rga5, which also increases Rho1 activity [26,33], did not stop septum ingression upon *bgs1* ^+^ repression (S3 Fig). Instead it partially attenuated the *bgs1* ^+^ repression phenotype which is consistent with the fact that Rho1 is the regulatory subunit of the β(1,3)glucan synthase enzyme. Therefore, the severe absence of septa caused by the lack of paxillin in Bgs1-depleted cells is likely not a direct consequence of Rho1 hyperactivation.

*bgs1* ^+^ repression leads to the formation of thick septa formed by layers of SS [11]. Because Ags1 and Bgs4 synthases are responsible for SS formation we analyzed the localization of these synthases in *pxl1Δ* cells with *bgs1* ^+^ repressed, as these cells are unable to form these septa. P*nmt81-bgs1* ^+^ and *pxl1*Δ P*nmt81-bgs1* ^+^ cells carrying Rlc1-RFP and either Ags1-GFP or GFP-Bgs4 were used. Upon *bgs1* ^+^ repression (24 h + T), both synthases appeared extended along the membrane of P*nmt81-bgs1* ^+^ cells. However, they still concentrated in the septum area where Rlc1-GFP formed a ring (Fig 3E, arrows) and in the growing poles. In contrast, in *pxl1*Δ P*nmt81-bgs1* ^+^ cells after 24 h of *bgs1* ^+^ repression Ags1 and Bgs4 were observed in the cytoplasm and along the plasma membrane (Fig 3E). These data suggest that Bgs1 and Pxl1 cooperate to maintain the synthases concentrated in the division area, and this is likely required for the synthesis of the septum structure.

**Fig 4:**
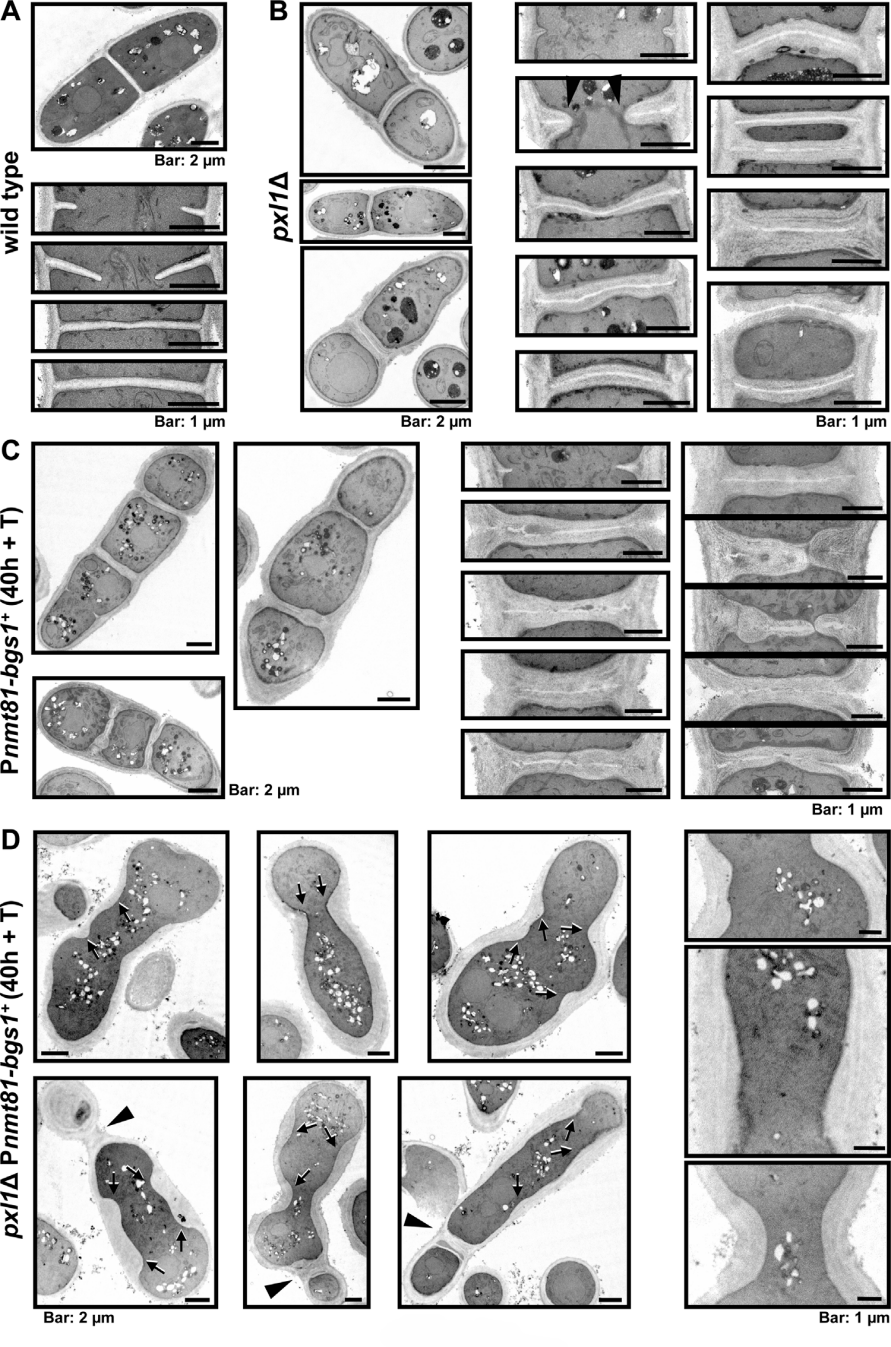
Bgs1 and Pxl1 cooperation is essential for the septum synthesis. **(A-D)** Transmission electron microscopy images of wild-type cells (A), *pxl1*Δ (B), P*nmt81-bgs1* ^+^ (C), and *pxl1*Δ P*nmt81-bgs1* ^+^ (D). Open arrowhead (in section B): Thick growing septum. Arrow (in section D): Projections of cell wall material that is laid down along the cell cortex. Arrowhead (in section D): Old thick septa without primary septum generated during earlier times of *bgs1* ^+^ repression. Cells were grown to early log-phase in EMM+S and shifted to EMM+S+T for 40 h, and then processed for electron microscopy as described in the Material and Methods section.

We performed transmission electron microscopy (TEM) to characterize in detail the cell wall of wild type, *pxl1*Δ, P*nmt81-bgs1* ^+^, and *pxl1*Δ P*nmt81-bgs1* ^+^ mutant cells cultured in EMM+S+T (Edinburgh Minimal Medium plus sorbitol and thiamine) during 40 h. Wild type cells showed a clear three-layered structure of PS flanked by two SS (Fig 4A). In cells lacking Pxl1 the septa were three-layered, but with thicker SS and cell walls (Fig 4B). Moreover, these thick structures were also observed in open septa, indicating that the absence of Pxl1 causes an excess of septum wall synthesis, forming thick SS structures from the early stages of septum ingression (Fig 4B, arrowhead). This Pxl1-dependent activation of wall synthesis could be caused by the increase in Rho1 activity described in *pxl1*Δ cells [26]. *bgs1* ^+^ repression generated multiseptated cells with aberrant septa totally different from those of wild type cells, as already described [11], and from those of *pxl1*Δ cells. The PS appeared discontinuous, twisted, or absent, and the septa were formed from the successive addition of SS layers parallel to the cell wall, which grew toward the cell center until a thick septum was generated ([11], and Fig 4C). Although aberrant, these thick septa progressed until completion. By contrast, the absence of Pxl1 during *bgs1* ^+^ repression caused an obvious absence of a cleavage furrow and septum synthesis. There was no defined membrane invagination and the cell wall grew inward over a large portion of the plasma membrane instead (Fig 4D). The deposited cell wall material pushed the plasma membrane toward the center of the cell along the longitudinal axis but no septa were completed (Fig 4D, arrow). A few very thick septa, probably formed during previous cell cycles with milder *bgs1* ^+^ repression, were observed (Fig 4D, arrowhead). The extended localization of Ags1 and Bgs4 might explain the thicker and non-uniform cell wall present in these cells (Fig 3E). Together, these results indicate that Pxl1 in the CAR and Bgs1 in the plasma membrane collaborate in the formation and ingression of the cleavage furrow.

### Cdc15 SH3 Domain Is Necessary for Proper Concentration of Pxl1 at the CAR

**Fig 5:**
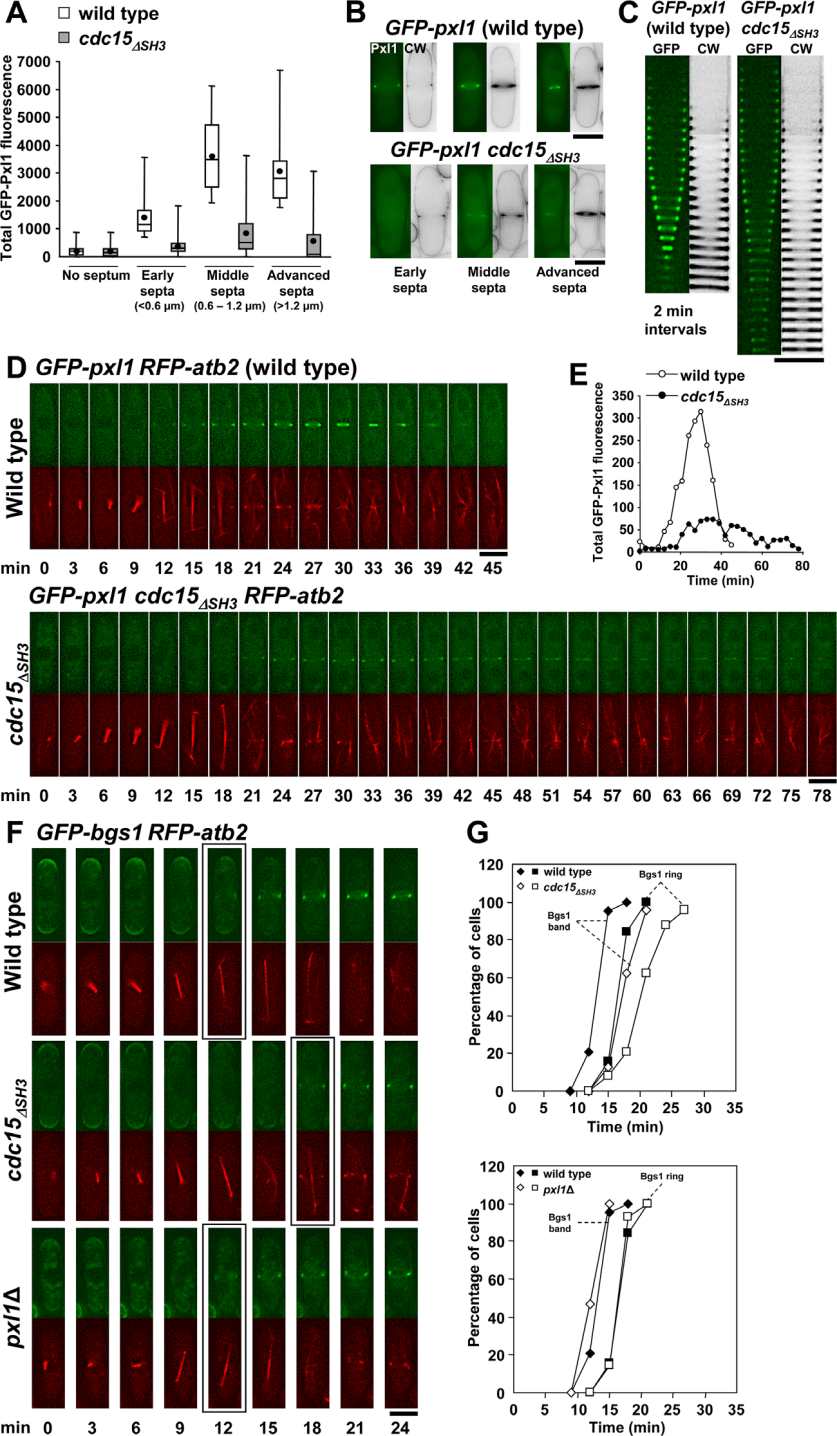
Cdc15 SH3 domain is necessary for proper concentration of Pxl1 at the CAR. **(A)** Box plot showing the total fluorescence of GFP-Pxl1 in the cell middle of wild-type (n = 90) and *cdc15*_ΔSH3_ cells (n = 214). GFP-Pxl1 fluorescence was measured in cells stained with CW, and divided into four categories depending on the length of the septum in the cell: 1) no septum 2) early septum (less than 0.6 μm); 3) middle septum (0.6 to 1.2 μm); and 4) advanced septum (more than 1.2 μm). Total fluorescence was quantified by using Image J software as described in the Materials and Methods section. **(B)** Fluorescence micrographs showing representative septated wild-type and *cdc15*_ΔSH3_ cells carrying GFP-Pxl1, and used to measure the total fluorescence of GFP-Pxl1 in A. **(C)** Kymographs of fluorescence time series (one middle z slide, 2 min intervals) of wild-type and *cdc15*_ΔSH3_ cells stained with CW and carrying GFP-Pxl1. **(D)** Time series of fluorescence micrographs (one medial z slide, 3 min intervals) of wild-type (upper panels) and *cdc15*_ΔSH3_ (lower panels) cells carrying GFP-Pxl1 and RFP-Atb2. Spindle microtubules appear at time 0. **(E)** Total fluorescence of GFP-Pxl1 in the cell middle of the time series shown in D. Fluorescence was quantified along the time as described in A. **(F)** Time series of fluorescence micrographs (one medial z slide, 3 min intervals) of cells carrying GFP-Bgs1 and RFP-Atb2. The first panel shows a wild-type cell where the Bgs1 band was detected in the septum assembly site at +12 min. The second panel shows a *cdc15*_ΔSH3_ cell where the Bgs1 band was detected in the septum assembly site at +18 min. The third panel shows a *pxl1*Δ cell where the Bgs1 band was detected in the septum assembly site at +12 min. Spindle microtubules appear at time 0. Squares indicate the first detection of Bgs1 as a band in the septum assembly site in the wild-type. **(G)** Time courses of appearance of the GFP-Bgs1 band (diamond) and GFP-Bgs1 ring (square). Open symbols are wild-type cells, and filled symbols are *cdc15*_ΔSH3_ (upper graph) and *pxl1*Δ (lower graph) cells. The same wild-type cells were used in both graphs. Wild-type (diamond n = 19; square n = 19), *cdc15*_ΔSH3_ (diamond n = 24; square n = 24), and *pxl1*Δ cells (diamond n = 15; square n = 14). Elapsed time is shown in minutes. Scale bars, 5 μm.

Pxl1 requires the SH3 domain of either Cdc15 or Imp2, two F-BAR proteins, to localize to the CAR [22]. Thus, Pxl1 localizes to the ring in cells carrying Cdc15_ΔSH3_ because it binds to Imp2. We observed that GFP-Pxl1 formed a ring in *cdc15*_*ΔSH3*_ cells but the fluorescence intensity was dimmer than in wild type cells (Fig 5A, 5B, 5C and 5D and S4A Fig, arrow). We quantified the GFP-Pxl1 ring fluorescence in wild type and *cdc15*_*ΔSH3*_ cells at four different times during cytokinesis: no septa, early septa (<0.6 μm), middle septa (0.6–1.2 μm), and advanced septa (>1.2 μm). This quantification showed that in wild type cells Pxl1 fluorescence increased from the onset of septation until the completion of the septum (Fig 5A and 5B). In contrast, no increase or even a reduction in GFP-Pxl1 fluorescence was observed in *cdc15*_*ΔSH3*_ cells (Fig 5A and 5B). Interestingly, reduction in Pxl1 fluorescence was coincident with a noticeably slower progression of the septum (Fig 5C). Time-lapse analysis performed in wild type and *cdc15*_*ΔSH3*_ cells carrying GFP-Pxl1 and RFP-Atb2 confirmed the results obtained in the quantification of septating cells (Fig 5D and 5E). In wild type cells the Pxl1 ring was detected at +12 min after the spindle appeared and began to constrict at +21 min while in *cdc15*_*ΔSH3*_ cells the Pxl1 ring was detected at +18 min and began to constrict at +39 min (Fig 5D). Moreover, GFP-Pxl1 intensity in *cdc15*_*ΔSH3*_ cells was never greater than 25% of the intensity in wild type cells (Fig 5E). These results suggest that an increase in Pxl1 might be necessary to start and complete septation efficiently.

The analysis of the cytokinetic defects caused by the lack of Cdc15 SH3 domain using *cdc15*_*ΔSH3*_ *-GFP rlc1* ^+^ *-RFP* cells revealed that in some cells with open septa Cdc15_ΔSH3_ delocalization and CAR disassembly occurred (S4B and S4C Fig), coinciding with a delocalization of Bgs1, which extended along the septum membrane, and a slower rate of septum formation (S4D Fig). Occasionally, the disappearance of Cdc15_ΔSH3_ was also accompanied with a reduced CW stain (S4C Fig, lower panels). This reduction might be caused by the recently described delay in the traffic of Bgs1 from the trans-Golgi network to the plasma membrane that occurs in *cdc15*_*ΔSH3*_ cells during cytokinesis [20]. To see if the decrease in Pxl1 was the cause of this delayed Bgs1 recruitment in *cdc15*_*ΔSH3*_ cells, we performed time-lapse experiments in wild type, *cdc15*_*ΔSH3*_, and *pxl1*Δ cells carrying GFP-Bgs1 and RFP-Atb2. Bgs1 was detected as a band in the septum assembly site at +12 min after the spindle appeared in wild type and also in *pxl1*Δ cells, while in *cdc15*_*ΔSH3*_ cells the Bgs1 band was detected at +18 min (Fig 5F and 5G). Time measurements of the appearance of GFP-Bgs1 as a ring confirmed the already described recruitment delay in *cdc15*_*ΔSH3*_ cells [20]. In contrast, no Bgs1 delay or reduction was detected in *pxl1*Δ cells (Fig 5F and 5G).

Taken together these results suggest that Cdc15 SH3 domain is necessary for Pxl1 recruitment to the CAR and for Bgs1 transport to the plasma membrane at the septum area as described [20,22] but that these processes do not depend on each other.

### Cooperation between Bgs1 and the SH3 Domain of Cdc15 Is Essential for CAR Maintenance and Septum Formation

**Fig 6:**
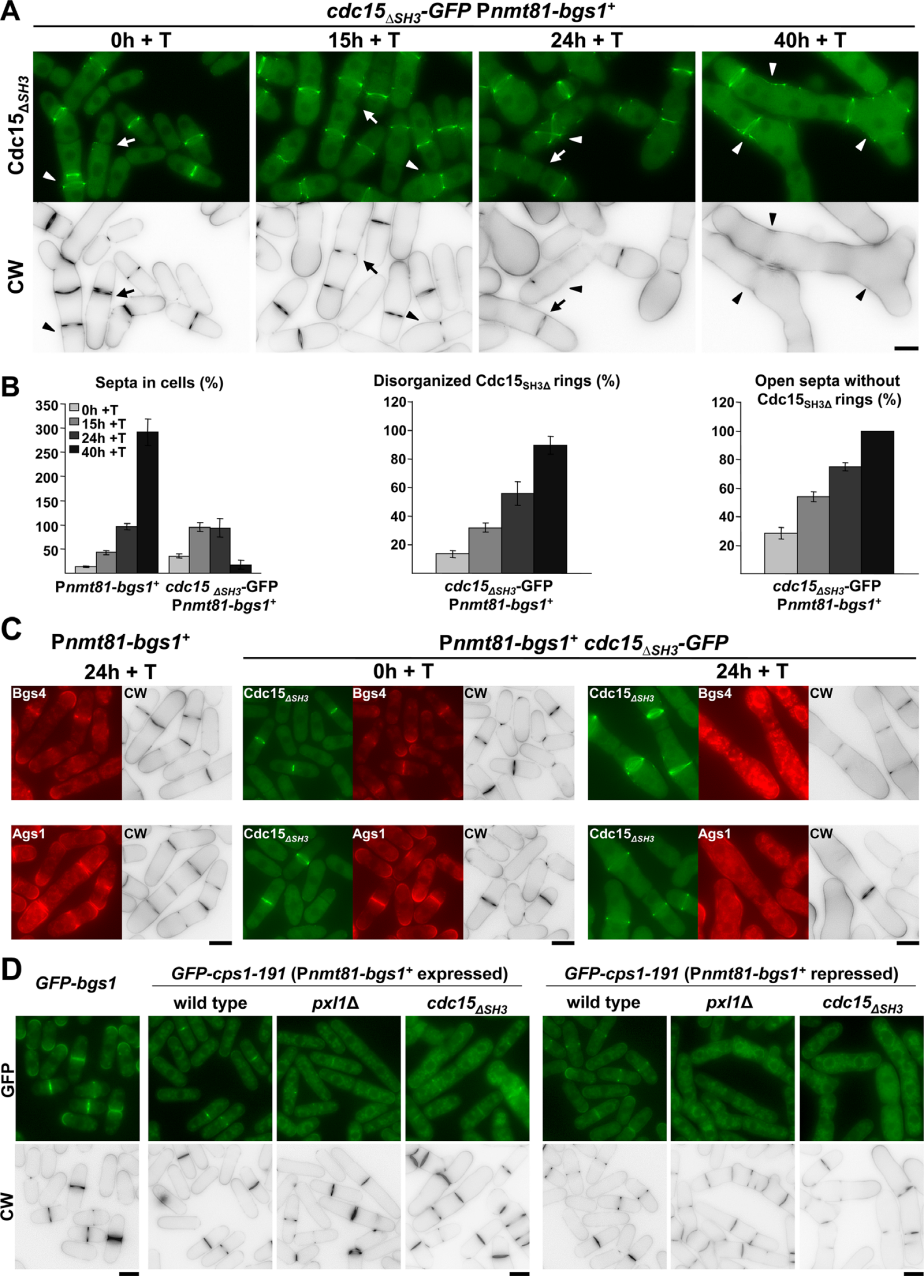
Cooperation between Bgs1 and the SH3 Domain of Cdc15 is essential for CAR maintenance and septum formation. **(A)** Fluorescence micrographs of *cdc15*_ΔSH3_-*GFP* P*nmt81-bgs1* ^+^ cells stained with CW and carrying Cdc15_ΔSH3_-GFP. Cells were grown to early log-phase in EMM+S (time 0 h), shifted to the same medium plus thiamine, EMM+S+T (times 15, 24 and 40 h, *bgs1* ^+^ repressed) and imaged at the indicated times. Arrow: Cell with an open septum without the ring of Cdc15. Arrowhead: Cell with a disorganized ring of Cdc15. **(B)** Histograms showing the indicated percentages of septa and Cdc15 structures in the strains P*nmt81-bgs1* ^+^ (n = 150 cells or hypha units were quantified for each time) and *cdc15*_ΔSH3_-*GFP* P*nmt81-bgs1* ^+^ (n = 100 cells or hypha units were quantified for each time). Note that P*nmt81-bgs1* ^+^ strains after 24 h of *bgs1* ^+^ repression appear as hypha units, each being equivalent to several single cells. (**C**) Fluorescence micrographs of P*nmt81-bgs1* ^+^ and *cdc15*_ΔSH3_-*GFP* P*nmt81-bgs1* ^+^ cells stained with CW and carrying Ags1-RFP or RFP-Bgs4 after the indicated times of repression of *bgs1* ^+^. **(D)** Fluorescence micrographs of P*nmt81-bgs1* ^+^, P*nmt81-bgs1* ^+^ *pxl1*Δ and P*nmt81-bgs1* ^+^ *cdc15*_ΔSH3_ cells carrying the hypomorphic version of Bgs1 GFP-Cps1-191, and wild-type cells carrying GFP-Bgs1 used as Bgs1 localization control. Since *cps1-191* allele is lethal in *pxl1*Δ and *cdc15*_ΔSH3_ backgrounds, strains are maintained alive with an inducible version of *bgs1* ^+^ (P*nmt81-bgs1* ^+^). Cells were grown at 25°C (permissive temperature for *cps1-191* cells) in EMM+S (time 0 h, *bgs1* ^+^ induced) and shifted to EMM+S+T (time 24 h +T, *bgs1* ^+^ repressed) to visualize cell phenotype and GFP-Cps1-191 localization. Scale bars, 5 μm.

As described above, Pxl1 cooperates with Bgs1 in the formation of the septum; therefore we investigated if Cdc15, through the SH3 domain, also cooperates with Bgs1 in septum formation. We made and analyzed the P*nmt81-bgs1* ^+^ *cdc15*_*ΔSH3*_ *-GFP* strain grown in EMM+S+T during different times (Fig 6A and 6B). The absence of the SH3 domain of Cdc15 mimicked the phenotype observed in the absence of Pxl1 during the repression of *bgs1* ^+^ (see Fig 3). An increase of cells with open septa without a CAR (analyzed as Cdc15_ΔSH3_-GFP ring) was observed during growth in the presence of thiamine (Fig 6A, arrow, and 6B), and after 48 h cells showed wide cell wall invaginations and no septum synthesis as detected by CW staining. The CAR of these cells was disorganized and did not constrict (Fig 6A and 6B). The localization of Ags1 and Bgs4 synthases was analyzed in P*nmt81-bgs1* ^+^ *cdc15*_*ΔSH3*_ *-GFP* cells carrying Ags1-RFP and RFP-Bgs4 upon *bgs1* ^+^ repression. As described in *pxl1*Δ P*nmt81-bgs1* ^+^ cells, after 24 h with thiamine, both synthases appeared in the cytoplasm and extended along the plasma membrane of P*nmt81-bgs1* ^+^ *cdc15*_*ΔSH3*_ *-GFP* cells (Fig 6C). Collectively, these results suggest that Cdc15, through the SH3 domain, and likely through Pxl1, cooperates with Bgs1 to confer stability to the CAR and to form the septum during cytokinesis.

To compare *pxl1*Δ and *cdc15*_*ΔSH3*_ phenotypes in relation to Bgs1 function and septum synthesis we tried to construct a *cdc15*_*ΔSH3*_ *cps1-191* strain, however these double mutant cells were nonviable. The microcolonies generated upon spore germination were similar to those formed by *pxl1*Δ *cps1-191* spores which are also nonviable (S5 Fig and [26]). We investigated the reason for this lethality by constructing wild type, *pxl1*Δ, and *cdc15*_*ΔSH3*_ strains carrying GFP-Cps1-191 in the presence of P*nmt81-bgs1* ^+^ to maintain the viability (Fig 6D). We analyze GFP-Cps1-191 localization before and after *bgs1* ^+^ repression at the permissive temperature. In the wild type background GFP-Cps1-191 was detected in the septum, either with *bgs1* ^+^ expressed or repressed, although less intense than GFP-Bgs1. In *pxl1*Δ and *cdc15*_*ΔSH3*_ cells Cps1-191 was detected in the septum area of cells expressing *bgs1* ^+^, but not after *bgs1* ^+^ repression. In this condition Cps1-191 was observed as fluorescent cytoplasmic aggregates, and as a weak fluorescence extended along the plasma membrane (Fig 6D, right panels). Thus, the faint GFP-Cps1-191 localization requires the support of functional wild type Bgs1 when Pxl1 or Cdc15 functions are compromised. These results might explain the lethality produced by *cps1-191* in *pxl1*Δ and *cdc15*_*ΔSH3*_, and support a role for Cdc15 and Pxl1 in the Bgs1 localization in the septum membrane, already described for Cdc15 [20] but newly observed for paxillin.

### A Joint Reduction of Cdc15 and Pxl1 Functions Induces Severe CAR Sliding and Causes Septum Material Deposition along the Plasma Membrane

**Fig 7:**
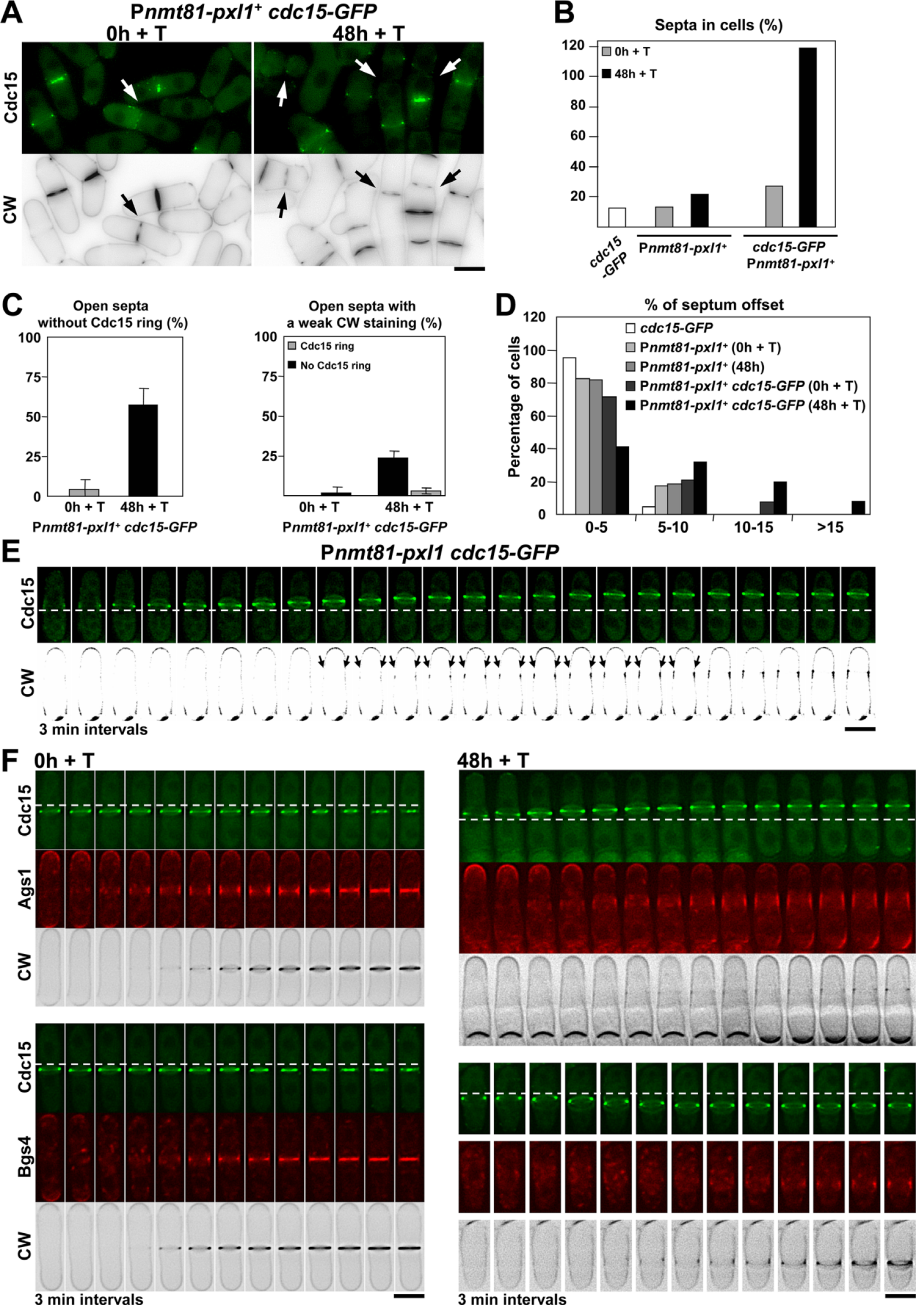
A joint reduction of Cdc15 and Pxl1 functions induces severe CAR sliding and causes septum material deposition along the plasma membrane. **(A)** Fluorescence micrographs of P*nmt81-pxl1* ^+^ cells stained with CW and carrying hypomorphic Cdc15-GFP. Early log-phase cells growing at 28°C in EMM+S (time 0 h) were shifted to EMM+S+T (time 48 h +T) to repress *pxl1* ^+^. Arrow: open septa without a Cdc15 ring and with lower CW staining. (**B)** Percentage of septa in *cdc15-GFP* (n = 840), P*nmt81-pxl1* ^+^ (at least 878 cells were quantified for each time) and *cdc15-GFP* P*nmt81-pxl1* ^+^ (at least 383 cells were quantified for each time). (**C)** Percentage of open septa without a stable Cdc15 ring (left) and open septa with a weak CW staining (right) in the strain P*nmt81-pxl1* ^+^ *cdc15-GFP* at the indicated times of *pxl1* ^+^ repression (at least 70 open septa were quantified for each time). **(D)** Histogram showing the indicated intervals of positions of the septa measured as the percent of septum offset from the cell center: white bars, *cdc15-GFP* cells (n = 42); light grey bars, P*nmt81-pxl1* ^+^ cells (time 0 h, *pxl1* ^+^ induced) (n = 51); middle grey bars, P*nmt81-pxl1* ^+^ cells (time 48 h + T, *pxl1* ^+^ repressed) (n = 60); dark grey bars, P*nmt81-pxl1* ^+^ *cdc15-GFP* cells (time 0 h, *pxl1* ^+^ induced) (n = 52); and black bars, P*nmt81-pxl1* ^+^ *cdc15-GFP* cells (time 48 h + T, *pxl1* ^+^ repressed) (n = 66). The percentages of septum offset were measured from CW staining images and calculated as is described for the Fig 1B. **(E)** Time series of fluorescence micrographs (one middle z slide, 3 min intervals) of P*nmt81-pxl1* ^+^ cells carrying hypomorphic Cdc15-GFP. Cells were grown at 28°C in EMM+S+T for 48 h (*pxl1* ^+^ repressed) and imaged. Arrow: Ring sliding and longitudinal synthesis of septum wall material along the cell cortex. For a better observation of longitudinal septum wall material synthesis, a high contrast has been applied to the CW images. **(F)** Time series of fluorescence micrographs (one middle z slide, 3 min intervals) of P*nmt81-pxl1* ^+^ cells carrying hypomorphic Cdc15-GFP and RFP-Bgs4 or Ags1-RFP. Cells were grown at 28°C in EMM+S in the absence of T (left, time 0 h, *pxl1* ^+^ induced) or shifted to EMM+S+T (right, time 48 h + T, *pxl1* ^+^ repressed) and imaged. Dashed line: reference for the ring position. Scale bars, 5 μm.

To further analyze the relationship between Pxl1 and Cdc15 we tried to construct a *pxl1*Δ *cdc15*_*ΔSH3*_ double mutant strain but it was not viable [34], supporting that *cdc15*_*ΔSH3*_ cytokinetic defects are not just caused by the lack of Pxl1, and that Cdc15 and Pxl1 might have additional functions that together are essential. A P*nmt41-pxl1* ^+^ *cdc15*_*ΔSH3*_ mutant strain was not viable either, even in the absence of thiamine when the *nmt41* promoter is active. Probably *pxl1* ^+^ expression level is critical in *cdc15*_*ΔSH3*_ mutant cells and P*nmt41-pxl1* ^+^ expression is not regulated by the transcription factor Ace2 during septation as it is the expression of the endogenous *pxl1* ^+^ [35]. GFP-tagging of Cdc15 at the C-terminus makes the protein partially nonfunctional, generating a hypomorphic allele also referred as *cdc15-gc1* [36]. Indeed, deletion of Pxl1 is lethal in *cdc15-GFP* cells [26]. Therefore, we made a P*nmt81-pxl1* ^+^ *cdc15-GFP* double mutant strain that was viable in the absence of thiamine (Fig 7A). Repression of *pxl1* ^+^ in these cells induced the accumulation of septa, and 30% of the cells became multiseptated after 48 h of *pxl1* ^+^ repression (Fig 7B). We also observed a considerable increase (5x) of open septa without a Cdc15-GFP ring and septa with a weak CW staining (60% and 30% after 48 h of *pxl1* ^+^ repression respectively; Fig 7A, arrow and 7C). In addition, *pxl1* ^+^ repression induced a significant increase in the number of *cdc15-GFP* cells with misplaced septa (Fig 7D), probably as a consequence of severe CAR sliding from the middle of the cell. This phenotype was also observed by time-lapse microscopy. While the CAR sliding in *pxl1*Δ cells stopped with septum synthesis, in P*nmt81-pxl1* ^+^ *cdc15*-*GFP* cells some CARs continued sliding even after the onset of septum synthesis, causing a longitudinal deposition along the plasma membrane of linear β-glucan as detected by CW staining until septum ingression started (Fig 7E, arrow).

To see if the other cell wall synthases also moved along with the CAR we performed time-lapse studies of CW-stained P*nmt81-pxl1 cdc15-GFP* cells carrying Ags1-GFP or GFP-Bgs4 at time 0 and after 48 h with thiamine (Fig 7F). Ags1 slide with the CAR and the CW-stained material along the cell membrane in the *pxl1* ^+^-repressed cells (Fig 7F, upper panels). In contrast, Bgs4 did not slide with the CAR but delayed its concentration at the septum assembly site in the cells depleted of *pxl1* (Fig 7F, lower panels).

Therefore we conclude that Pxl1 and Cdc15 collaborate in CAR and Bgs1 stability. More important, Pxl1 and Cdc15 cooperate to couple CAR contraction with the onset of septum synthesis and cleavage furrow formation. Uncoupling both processes results in the synthesis of septum wall material along the plasma membrane.

## Discussion

Fission yeast Pxl1 binds to the type II myosin Myo2 and the F-BAR protein Cdc15, and participates in maintaining CAR integrity [26]. We show in this work that Pxl1 also collaborates in the stable anchorage of the ring to the membrane since the absence of Pxl1 causes CAR sliding until the onset of septation. The extent of CAR sliding in *pxl1*Δ strain was similar to that described in *cdc15*_*ΔSH3*_ cells [20], with more than 10% offset septa in around 30% of the cells. It is possible that Pxl1 participates in the physical link between the CAR and the plasma membrane mediated through Cdc15, which has been shown to play a role in this linkage [22]. It has recently been proposed that Bgs1 protein, besides being a major enzyme for PS formation, is the physical anchor of the ring to the plasma membrane and that Cdc15 participates in the transfer of Bgs1 from the Golgi apparatus to the plasma membrane [20]. We show here that the septum synthases Bgs1 and Ags1, located at the ring edge membrane, slide together with the CAR in cells lacking Pxl1, indicating that these transmembrane enzymes are connected to the CAR but are not sufficient to anchor it stably. Instead, the coincidence between the appearance of CW staining and the cessation of ring sliding, which was also shown in cells depleted of Bgs4 [4], suggest that the PS synthesized by Bgs1 is required for the CAR to be stably anchored. Corroborating this hypothesis, *bgs1* ^+^ mutations, such as that of *cps1-191* allele [37], permit CAR sliding [20]. In addition, our results indicate that defective PS synthesis and ingression induces CAR instability, and that collaboration of Bgs1 with Pxl1 and the SH3 domain of Cdc15 is required to maintain the CAR structure and to form the septum, globally pointing to the existence of a physical link between the CAR and the PS through Pxl1, Cdc15, and Bgs1.

In the budding yeast *S*. *cerevisiae* CAR closure and formation of the PS are processes that depend on one another. Thus, interference with one function would also block the other [38]. Mutant cells lacking either myosin type II or the chitin synthase Chs2, which is responsible for making the PS in this yeast, have indistinguishable cytokinesis defects and the double mutant lacking both myosin type II and Chs2 hardly differs from the single ones. Similarly, in fission yeast defects in the CAR can alter the rate and symmetry of septum ingression [3,39], and vice versa [37]. However, in contrast with budding yeast, the type II myosin Myo2 is essential in fission yeast. Additionally, we show here that simultaneous depletion of Pxl1 and alteration of Cdc15 function uncouples two tightly linked processes, CAR closure and PS formation, resulting in the synthesis of CW-stained material along the plasma membrane advancing with a sliding CAR that is unable to constrict. When Bgs1 is depleted, Pxl1 is required for the salvage route that leads to the abnormal but complete septa found in these cells. The remedial septa under Bgs1 depletion are formed by layers of the SS [11], which somehow are guided by the CAR. It is possible that Pxl1 participates in the transmission of the ring tension generated by myosin II to the membrane in order to concentrate the SS synthases and narrow the area of septum synthesis. In the absence of Bgs1 and Pxl1 there is no PS synthesis and the connection between the plasma membrane and the ring is lost. As a consequence, there is no concentration of the SS synthases and some inward growth of cell wall along the lateral plasma membrane occurs, but cytokinesis is never accomplished.

At present it is not clear how Pxl1 contributes to Bgs synthases function. It is tempting to propose that Pxl1 is acting as a mechanosensor that helps to transform the CAR contraction into an activation signal for the biosynthetic enzymes that form the septum. In this sense Pxl1 interacts with Rho1 GTPase, the major activator of both β(1,3)glucan synthase catalytic subunits [9,26]. F-BAR proteins present at the ring edge membrane such as Cdc15, Imp2, or the recently described Rga7 might be part of this transmission [22,23]. In fact, Cdc15_ΔSH3_ causes defects similar to those caused by the lack of paxillin in Bgs1-depleted cells, suggesting that they are part of the same signaling pathway. Paxillin might collaborate with Cdc15 in the transfer of Bgs1 from the Golgi to the plasma membrane [20]. However, no delay in the transport of this synthase to the division site membrane was observed in cells lacking Pxl1. On the other hand, GFP-tagged Cps1-191 localizes to the membrane in wild type cells but forms intracellular aggregates in both *pxl1*Δ and *cdc15*_*ΔSH3*_. Importantly, these aggregates are not observed in *pxl1*Δ or *cdc15*_*ΔSH3*_ cells carrying wild type Bgs1, suggesting that the correct function of Bgs1 helps its localization at the plasma membrane. The observed aggregates in *pxl1*Δ or *cdc15*_*ΔSH3*_ cells could be caused by a deficient transport and/or recycling of nonfunctional Cps1-191 from trans-Golgi or endosomes to the plasma membrane.

The lack of paxillin is lethal in cells carrying *cdc15*_*ΔSH3*_ or even the hypomorphic allele *cdc15-GFP* [26,34]. As we show here, Cdc15 is necessary to concentrate Pxl1 to the CAR but is also required to concentrate Bgs1 to the membrane [20]. This double function could explain the lethality of *pxl1*Δ *cdc15*_*ΔSH3*_ double mutation. It also could explain the observed phenotype in *cdc15-GFP* hypomorphic mutant cells depleted of Pxl1. CAR sliding in these double mutant cells is more pronounced and longitudinal synthesis of linear β(1,3)glucan without ring contraction is sometimes observed, as if a low concentration of active Bgs1 was not sufficient to anchor the defective CAR of Pxl1-depleted cells, nor to activate CAR contraction and septum ingression.

The lack of paxillin is also lethal in the absence of other F-BAR proteins such as Rga7 [23]. Moreover, Pxl1 is essential in the absence of the C2-domain containing protein Fic1 which also binds to Cdc15 and Imp2 [22]. *S*. *cerevisiae* Inn1, which is the ortholog of Fic1, couples CAR constriction and plasma membrane ingression [22,40]. It has been proposed that Inn1 acts in conjunction with Hof1, the Cdc15 ortholog of budding yeast, and Cyk3 to regulate the catalytic domain of the PS-forming enzyme Chs2 [41]. Cyk3 and Chs2 orthologs exist in fission yeast and have a role in cytokinesis [42,43]. However, the lack of Fic1, Cyk3, or Chs2 orthologs does not cause a drastic phenotype in *S*. *pombe* cells, which do not have chitin in the septum wall. It will be interesting to determine whether Fic1, like Inn1, is required for CAR closure during cytokinesis.

In summary, our studies indicate that both CAR and PS collaborate in the cleavage furrow and septum formation, shedding some light on the importance of the connection between the ring and the septum during *S*. *pombe* cytokinesis.

## Materials and Methods

### Strains, Growth Conditions, and Genetic Methods

The *S*. *pombe* strains used in this study are enumerated in S1 Table.

*pxl1*Δ deletions by replacement with the *KanMX6* or *ura4* ^+^ gene and *GFP-pxl1* ^+^ strains have been described [26]. P*nmt41*-*pxl1* ^+^ and P*nmt81*-*pxl1* ^+^ strains contain *pxl1* ^+^ expressed under the control of the medium and low expression versions respectively of the thiamine-repressible *nmt1* ^+^ promoter and were obtained by PCR-based gene targeting as described [44].

Strains 435 and 526 contain *bgs1*Δ::*ura4* ^+^ deletion and the multicopy plasmids p81XH-*bgs1* ^+^ and p81XL-*bgs1* ^+^ (*his3* ^+^ and *LEU2* selection, respectively), which contain *bgs1* ^+^ expressed under the control of the *nmt81* version of the thiamine-repressible *nmt1* ^+^ promoter [31]. P*nmt81*-*bgs1* ^+^ strain 1483 contains the selection marker *ura4* ^+^ adjacent to the P*nmt81* promoter, followed by the *bgs1* ^+^ ORF. This strain was made from a diploid strain by homologous recombination of an ApaI-NotI P*bgs1* ^+^-*ura4* ^+^-81X-*bgs1* (nt 1–982) fragment (see below) and sporulation. The resulting P*nmt81*-*bgs1* ^+^ haploid strain contained one single integrated *bgs1* ^+^ copy under the control of the modified P*nmt81* promoter. The strains 435, 526 and 1483 exhibited a phenotype of elongated, branched and multiseptated cells in the presence of thiamine and a wild type phenotype in its absence as described before [11]. Other P*nmt81*-*bgs1* ^+^ strains were made by genetic cross and random spore analysis selecting against the corresponding parental auxotrophies.

*GFP-12A-cps1-191* P*nmt81*-*bgs1* ^+^ strain (5186) was made by transforming a *leu1-32* P*nmt81*-*bgs1* ^+^ strain with the integrative plasmid pJK-*GFP-12A-cps1-191* cut with Eco47III, which directed its integration to the *leu1-32* locus. In the absence of thiamine (*bgs1* ^+^ copy induced) this strain exhibited a wild type appearance at both 25°C and 36°C, while in the presence of thiamine (*bgs1* ^+^ copy repressed) this strain exhibited a wild type appearance at 25°C and a phenotype of arrested cells without or with a single septum at 36°C, as described before [37]. *GFP-12A-bgs1* ^+^ strain 1723 contain an integrated copy of SmaI-cut pJK-*GFP-12A-bgs1* ^+^ (*leu1* ^+^ selection), which directed its integration at the SmaI site adjacent to *bgs1*Δ::*ura4* ^+^, at position -748 of the *bgs1* ^+^ promoter sequence. Similarly, *tdTom-12A-bgs1* ^+^ strain 1780 contains integrated SmaI-cut pJK-*tdTom-12A-bgs1* ^+^ (tandem dimer Tomato variant of mRFP, [45]) at position -748 of the *bgs1* ^+^ promoter sequence. *GFP-bgs4* ^+^ and *ags1* ^+^ *-GFP* strains 562 and 3167 respectively, have already been described [12,15].

Standard *S*. *pombe* media and genetic manipulations were used [46]. Cells were grown either in rich medium (YES) or in minimal medium (EMM) with appropriate supplements. EMM+S (1.3M sorbitol) was used with P*nmt81*-*bgs1* ^+^ and P*nmt81*-*pxl1* ^+^, and derived strains. For repression experiments in strains carrying the P*nmt81* promoters, early log-phase cells incubated in EMM+S were diluted with the same medium plus 20 μg/ml thiamine. SPA medium was used for genetic crosses and mutant strains were selected by either tetrad dissection, random spore dissection or random spore germination methods. Cell growth was monitored by measuring the A_600_ of early-log phase cell cultures.

### Plasmids and Recombinant DNA Methods

pKS-*tdTomato* is pKS^+^ with a BamHI—EcoRI fragment from pRSET-*tdTomato* containing the 1,425-bp coding sequence of two tandem copies of the Tomato variant of the monomeric *mRFP1* (provided by R. Tsien, University of California at San Diego, La Jolla, CA, [45]). A 12-alanine coding sequence used as connector to provide flexibility was fused to the *RFP* 3´-end coding sequence of pKS-*tdTomato*, making pKS-*tdTom-12A*.

pJK-*GFP-12A-bgs1* ^+^, and pJK-*GFP-12A-bgs4* ^+^ are pJK-*bgs1* ^+^ and pJK-*bgs4* ^+^ with *GFP-12A* inserted in-frame after the start codon, at base 4 (amino acid 2) of the coding sequence. pJK-*tdTom-12A-bgs1* ^+^ is pJK-*bgs1* ^+^ with *tdTom-12A* inserted in-frame at base 4 of *bgs1* ^+^ coding sequence. Similarly, pJK-*ags1(1–6267)-12A-GFP-12A* and pJK-*ags1(1–6267)-8A-Cher-12A* are pJK-*ags1(1–6267)* with *12A-GFP-12A* and *8A-Cher-12A* inserted in-frame at base 5866 (amino acid 1956) of *ags1* ^+^ coding sequence.

p81XL-*bgs1* ^+^ and p81XH-*bgs1* ^+^ have been described previously [11].

pSK-P*bgs1* ^+^-*ura4* ^+^-81X-*bgs1(1–982)* contains a *bgs1* ^+^ promoter fragment (-2070 to -1088), the *ura4* ^+^ sequence and an P*nmt81-bgs1* ^+^ 5´ORF fragment (nt 1 to 982) from p81XH-*bgs1* ^+^. An ApaI-NotI P*bgs1* ^+^-*ura4* ^+^-81X-*bgs1(1–2603)* fragment was used as *bgs1* ^+^ substitution module to make the integrated P*nmt81-bgs1* ^+^ strains.

pJK-*GFP-12A-bgs1* ^*cps1-191*^ is pJK-*GFP-12A-bgs1* ^+^ with the codon 277 changed from GAT to AAT by site-directed mutagenesis [47], resulting in the D277N replacement as described in the *cps1-191* mutation [37].

All DNA manipulations were carried out by established methods [48]. Enzymes were used according to the recommendations of the suppliers. Plasmid DNA was introduced into *S*. *pombe* cells by an improved LiAc method [49]. Escherichia coli DH5αF´ was used as host to propagate plasmids by growth at 37°C in Luria-Bertani medium plus 50 μg/ml ampicillin.

### Microscopy Techniques and Data Analysis

Images from germinating spores (S5 Fig) were obtained directly from the growing plates with a Nikon Eclipse 50i microscope, a Nikon Plan FLUOR 20×/0.45 objective, a Nikon Ds-Fi1 digital camera and a Nikon Digital Sight DS-L2 control unit. For Calcofluor white labeling, a solution of Calcofluor white (CW; 50 μg/ml final concentration) was added directly to early logarithmic phase cells. Fluorescence Images were obtained with a Leica DM RXA fluorescence microscope, a PL APO 63×/1.32 OIL PH3 objective, a Leica DFC350FX digital camera and Leica CW4000 cytoFISH software. Images were processed with Adobe Photoshop software. CW images are shown as negative images to improve the fluorescence resolution.

Time-lapse imaging was performed as described in [15]. Early logarithmic phase cells were suspended in 0.3 ml of the corresponding growing medium containing CW (5 μg/ml final concentration) when necessary, and placed in a well from a μ-Slide 8 well or a μ-Slide 8 well glass bottom (80821-Uncoated and 80827; Ibidi) previously coated with 5 μl of 1 mg/ml soybean lectin (L1395; Sigma-Aldrich). All the time-lapse experiments were made at 28°C, with the exception of those showed in the Fig 2 which were imaged at 37°C, by acquiring epifluorescence and/or phase contrast cell images in single planes and 1×1 binning on an inverted microscope (Olympus IX71) equipped with a PlanApo 100x/1.40 IX70 objective and a Personal DeltaVision system (Applied Precision). Images were captured using CoolSnap HQ2 monochrome camera (Photometrics) and softWoRx 5.5.0 release 6 imaging software (Applied Precision). Subsequently, GFP and RFP time-lapse images were restored and corrected by 3D Deconvolution (conservative ratio, 10 iterations and medium noise filtering) through softWoRx imaging software. Next, images were processed with Image J (National Institutes of Health) and Adobe Photoshop softwares.

For maximal projection of the cell and three-dimensional reconstructions of the cell middle region in Fig 2A, 2B, 2F and 2G images were obtained in Z-stacks of 24 to 28 slices at 0.3 μm intervals to ensure that the complete cell is covered. Then only the slides that detected the cells were processed with the function stacks and 3D projection of the Image J software.

Total fluorescence analysis of a single focal plane of GFP-Pxl1 in Fig 5A and 5E was quantified with Image J software by selecting the middle region of each cell containing the ring. Next the background fluorescence of each cell was corrected through the Background subtraction from ROI function (Image J), and finally the sum of the remaining values of the pixels in the selected rectangle was obtained. After quantification of the GFP-Pxl1 fluorescence, the length of the corresponding septum was measured by drawing a line through the septum in the CW-staining micrograph. The results are represented in the box plot shown in Fig 5A, where the upper and lower lines of the box indicate the upper and lower quartiles, while the "whiskers" show the values furthest away from the median on either side of the box. The line inside the box is the middle-value (median) and the black dot is the mean.

Line scans in the S4C Fig were made by drawing a line along all the septum length, measuring the intensities of pixels of the Rlc1-RFP, Cdc15_ΔSH3_-GFP and CW stain fluorescences through the plot profile function of the Image J software.

The positions of the offset septa in Figs 1B, 3B and 7D were calculated from CW-stained images with the Image J software by measuring the distance from the septum to the closest tip, subtracting this value from the value corresponding to the half of the cell length, and calculating the percentage of the resulting value respect to the total cell length.

### Transmission Electron Microscopy

Early logarithmic phase cells were fixed with 2% glutaraldehyde EM (GA; Electron Microscopy Science) in 50 mM phosphate buffer pH 7.2, 150 mM NaCl (PBS) for 2 h at 4°C, post-fixed with 1.2% potassium permanganate overnight at 4°C and embedded in Quetol 812 as described [50]. Ultrathin sections were stained in 4% uranyl acetate and 0.4% lead citrate, and viewed with TEM H-800 (Hitachi) operating at 125 kV.

## Supporting Information

S1 Fig Pxl1 is required for stable Rlc1 ring positioning in the cell middle until septation onset. **(A)** Time series of fluorescence micrographs (one medial z slide, 5 min intervals) of *pxl1*Δ cells stained with CW and expressing Rlc1-RFP. Arrowheads: septation onset. Dashed line: reference for the ring position in the cell in each micrograph. **(B)** CW staining images of *pxl1*Δ cells expressing Rlc1-RFP (left) and GFP-Bgs1 and Rlc1-RFP (right). The graph indicates the percentage of septated (single septum) and multiseptated cells in *pxl1*Δ *rlc1-RFP* and *pxl1*Δ *rlc1-RFP GFP-bgs1* ^+^ (at least n = 390 cells were quantified for each strain). **(C)** Time series of fluorescence micrographs (one medial z slide, 2 min intervals) of wild type cells stained with CW and expressing Ags1-GFP. **(D)** Time series of fluorescence micrographs (one medial z slide, 3 min intervals) of *pxl1*Δ cells stained with CW, and expressing Ags1-GFP and Rlc1-RFP. Dashed line: reference for the ring position in the cell in each micrograph. Scale bars, 5 μm.
(TIF)

S2 Fig Pxl1 remains localized to the Rlc1 ring during Bgs1 depletion. **(A)** Fluorescence micrographs of P*nmt81-bgs1* ^+^ cells stained with CW and expressing GFP-Pxl1. Cells were grown in EMM+S (time 0 h), shifted to EMM+S+T for *bgs1* ^+^ repression (times 24 and 40 h + T), and imaged at the indicated times. Scale bar, 5 μm.
(TIF)

S3 Fig An increase in Rho1 activity does not mimic the absence of Pxl1 during *bgs1* ^+^ repression. **(A)** Phase contrast and fluorescence micrographs of *rga5*Δ, P*nmt81-bgs1* ^+^ and *rga5*Δ P*nmt81-bgs1* ^+^ cells stained with CW. Cells were grown in EMM+S (time 0 h), shifted to EMM+S+T for *bgs1* ^+^ repression (times 24 and 40 h + T), and imaged at the indicated times. Scale bars, 5 μm.
(TIF)

S4 Fig The absence of the SH3 domain of Cdc15 induces the loss of the ring in open septa and extension of Bgs1 from the septum edge along the septum membrane. **(A)** Fluorescence micrographs of wild type and *cdc15*_*ΔSH3*_ cells stained with CW and expressing GFP-Pxl1. Arrows: Reduction of GFP-Pxl1 fluorescence in open septa. **(B)** Fluorescence micrographs of *cdc15*_*ΔSH3*_ cells stained with CW and expressing Cdc15_ΔSH3_-GFP and Rlc1-RFP. Arrows: Absence of Cdc15 and Rlc1 rings in open septa. **(C)** Line scans showing the fluorescence intensity of Rlc1-RFP, Cdc15_ΔSH3_-GFP and CW along open septa in *cdc15*_*ΔSH3*_ cells. The x-axis represents distance along the septum line and the y-axis is the pixel intensity. Scans were made as described in the Material and Methods section. **(D)** Kymographs of fluorescence time series (one middle z slide, 2 min intervals) of *cdc15-GFP* and *cdc15*_ΔSH3_ *-GFP* cells expressing RFP-Bgs1. Scale bars, 5 μm.
(TIF)

S5 Fig A reduction of Bgs1 function induces lethality in *cdc15*_*ΔSH3*_ and *pxl1*Δ cells. **(A)** *cps1-191* cells were crossed with either *cdc15*_ΔSH3_-*FLAG* or *pxl1*Δ cells and tetrads were dissected. Colonies were imaged directly from the plate after 2 (viable colonies) and 6 days (non-viable colonies) of growth in YES plates at 25°C. Broken rectangles indicate non-viable double mutant colonies. Scale bars, 20 μm.
(TIF)

S1 Table *S*. *pombe* strains used in this study. (DOC)

## References

[1] Pollard TD, Wu JQ (2010) Understanding cytokinesis: lessons from fission yeast. Nat Rev Mol Cell Biol 11: 149–155. 10.1038/nrm2834 20094054 PMC2819279

[2] Balasubramanian MK, Srinivasan R, Huang Y, Ng KH (2012) Comparing contractile apparatus-driven cytokinesis mechanisms across kingdoms. Cytoskeleton (Hoboken) 69: 942–956. 23027576 10.1002/cm.21082

[3] Proctor SA, Minc N, Boudaoud A, Chang F (2012) Contributions of turgor pressure, the contractile ring, and septum assembly to forces in cytokinesis in fission yeast. Curr Biol 22: 1601–1608. 10.1016/j.cub.2012.06.042 22840513 PMC3443309

[4] Muñoz J, Cortés JCG, Sipiczki M, Ramos M, Clemente-Ramos JA, et al (2013) Extracellular cell wall β(1,3)glucan is required to couple septation to actomyosin ring contraction. J Cell Biol 203: 265–282. 10.1083/jcb.201304132 24165938 PMC3812973

[5] Wu JQ, Kuhn JR, Kovar DR, Pollard TD (2003) Spatial and temporal pathway for assembly and constriction of the contractile ring in fission yeast cytokinesis. Dev Cell 5: 723–734. 14602073 10.1016/s1534-5807(03)00324-1

[6] Balasubramanian MK, Bi E, Glotzer M (2004) Comparative analysis of cytokinesis in budding yeast, fission yeast and animal cells. Curr Biol 14: R806–818. 15380095 10.1016/j.cub.2004.09.022

[7] Rincon SA, Paoletti A (2012) Mid1/anillin and the spatial regulation of cytokinesis in fission yeast. Cytoskeleton (Hoboken) 69: 764–777. 22888038 10.1002/cm.21056

[8] Lee IJ, Coffman VC, Wu JQ (2012) Contractile-ring assembly in fission yeast cytokinesis: Recent advances and new perspectives. Cytoskeleton (Hoboken) 69: 751–763. 22887981 10.1002/cm.21052 PMC5322539

[9] Arellano M, Durán A, Pérez P (1996) Rho 1 GTPase activates the (1–3)beta-D-glucan synthase and is involved in *Schizosaccharomyces pombe* morphogenesis. EMBO J 15: 4584–4591. 8887550 PMC452188

[10] Drgonova J, Drgon T, Tanaka K, Kollar R, Chen GC, et al (1996) Rho1p, a yeast protein at the interface between cell polarization and morphogenesis. Science 272: 277–279. 8602514 10.1126/science.272.5259.277

[11] Cortés JCG, Konomi M, Martins IM, Muñoz J, Moreno MB, et al (2007) The (1,3)β-D-glucan synthase subunit Bgs1p is responsible for the fission yeast primary septum formation. Mol Microbiol 65: 201–217. 17581129 10.1111/j.1365-2958.2007.05784.x

[12] Cortés JCG, Carnero E, Ishiguro J, Sanchez Y, Durán A, et al (2005) The novel fission yeast (1,3)β-D-glucan synthase catalytic subunit Bgs4p is essential during both cytokinesis and polarized growth. J Cell Sci 118: 157–174. 15615781 10.1242/jcs.01585

[13] Hochstenbach F, Klis FM, van den Ende H, van Donselaar E, Peters PJ, et al (1998) Identification of a putative alpha-glucan synthase essential for cell wall construction and morphogenesis in fission yeast. Proc Natl Acad Sci U S A 95: 9161–9166. 9689051 10.1073/pnas.95.16.9161 PMC21309

[14] Katayama S, Hirata D, Arellano M, Pérez P, Toda T (1999) Fission yeast α-glucan synthase Mok1 requires the actin cytoskeleton to localize the sites of growth and plays an essential role in cell morphogenesis downstream of protein kinase C function. J Cell Biol 144: 1173–1186. 10087262 10.1083/jcb.144.6.1173 PMC2150588

[15] Cortés JCG, Sato M, Muñoz J, Moreno MB, Clemente-Ramos JA, et al (2012) Fission yeast Ags1 confers the essential septum strength needed for safe gradual cell abscission. J Cell Biol 198: 637–656. 10.1083/jcb.201202015 22891259 PMC3514033

[16] Wolfe BA, Gould KL (2005) Split decisions: coordinating cytokinesis in yeast. Trends Cell Biol 15: 10–18. 15653073 10.1016/j.tcb.2004.11.006

[17] Carnahan RH, Gould KL (2003) The PCH family protein, Cdc15p, recruits two F-actin nucleation pathways to coordinate cytokinetic actin ring formation in *Schizosaccharomyces pombe* . J Cell Biol 162: 851–862. 12939254 10.1083/jcb.200305012 PMC2172828

[18] Roberts-Galbraith RH, Ohi MD, Ballif BA, Chen JS, McLeod I, et al (2010) Dephosphorylation of F-BAR protein Cdc15 modulates its conformation and stimulates its scaffolding activity at the cell division site. Mol Cell 39: 86–99. 10.1016/j.molcel.2010.06.012 20603077 PMC2916701

[19] Arasada R, Pollard TD (2011) Distinct roles for F-BAR proteins Cdc15p and Bzz1p in actin polymerization at sites of endocytosis in fission yeast. Curr Biol 21: 1450–1459. 10.1016/j.cub.2011.07.046 21885283 PMC3350781

[20] Arasada R, Pollard TD (2014) Contractile Ring Stability in S. pombe Depends on F-BAR Protein Cdc15p and Bgs1p Transport from the Golgi Complex. Cell Rep 8: 1533–1544. 10.1016/j.celrep.2014.07.048 25159149 PMC4163078

[21] Demeter J, Sazer S (1998) imp2, a new component of the actin ring in the fission yeast *Schizosaccharomyces pombe* . J Cell Biol 143: 415–427. 9786952 10.1083/jcb.143.2.415 PMC2132827

[22] Roberts-Galbraith RH, Chen JS, Wang J, Gould KL (2009) The SH3 domains of two PCH family members cooperate in assembly of the *Schizosaccharomyces pombe* contractile ring. J Cell Biol 184: 113–127. 10.1083/jcb.200806044 19139265 PMC2615086

[23] Martín-Garcia R, Coll PM, Pérez P (2014) F-BAR domain protein Rga7 collaborates with Cdc15 and Imp2 to ensure proper cytokinesis in fission yeast. J Cell Sci. 10.1242/jcs.146233 25052092

[24] Kume K, Kubota S, Koyano T, Kanai M, Mizunuma M, et al (2013) Fission yeast leucine-rich repeat protein Lrp1 is essential for cell morphogenesis as a component of the morphogenesis Orb6 network (MOR). Biosci Biotechnol Biochem 77: 1086–1091. 23649273 10.1271/bbb.130064

[25] Ge W, Balasubramanian MK (2008) Pxl1p, a paxillin-related protein, stabilizes the actomyosin ring during cytokinesis in fission yeast. Mol Biol Cell 19: 1680–1692. 10.1091/mbc.E07-07-0715 18272786 PMC2291416

[26] Pinar M, Coll PM, Rincón SA, Pérez P (2008) *Schizosaccharomyces pombe* Pxl1 is a paxillin homologue that modulates Rho1 activity and participates in cytokinesis. Mol Biol Cell 19: 1727–1738. 10.1091/mbc.E07-07-0718 18256290 PMC2291433

[27] Cortés JCG, Ishiguro J, Durán A, Ribas JC (2002) Localization of the (1,3)β-D-glucan synthase catalytic subunit homologue Bgs1p/Cps1p from fission yeast suggests that it is involved in septation, polarized growth, mating, spore wall formation and spore germination. J Cell Sci 115: 4081–4096. 12356913 10.1242/jcs.00085

[28] Tebbs IR, Pollard TD (2013) Separate roles of IQGAP Rng2p in forming and constricting the *Schizosaccharomyces pombe* cytokinetic contractile ring. Mol Biol Cell 24: 1904–1917. 10.1091/mbc.E12-10-0775 23615450 PMC3681696

[29] Nakamura T, Nakamura-Kubo M, Hirata A, Shimoda C (2001) The Schizosaccharomyces pombe spo3+ gene is required for assembly of the forespore membrane and genetically interacts with psy1(+)-encoding syntaxin-like protein. Mol Biol Cell 12: 3955–3972. 11739793 10.1091/mbc.12.12.3955 PMC60768

[30] Forsburg SL (1993) Comparison of *Schizosaccharomyces pombe* expression systems. Nucleic Acids Res 21: 2955–2956. 8332516 10.1093/nar/21.12.2955 PMC309706

[31] Moreno MB, Durán A, Ribas JC (2000) A family of multifunctional thiamine-repressible expression vectors for fission yeast. Yeast 16: 861–872. 10861909 10.1002/1097-0061(20000630)16:9<861::AID-YEA577>3.0.CO;2-9

[32] Onishi M, Ko N, Nishihama R, Pringle JR (2013) Distinct roles of Rho1, Cdc42, and Cyk3 in septum formation and abscission during yeast cytokinesis. J Cell Biol 202: 311–329. 10.1083/jcb.201302001 23878277 PMC3718969

[33] Calonge TM, Arellano M, Coll PM, Pérez P (2003) Rga5p is a specific Rho1p GTPase-activating protein that regulates cell integrity in *Schizosaccharomyces pombe* . Mol Microbiol 47: 507–518. 12519200 10.1046/j.1365-2958.2003.03312.x

[34] Ren L, Willet AH, Roberts-Galbraith RH, McDonald NA, Feoktistova A, et al (2015) The Cdc15 and Imp2 SH3 domains cooperatively scaffold a network of proteins that redundantly ensure efficient cell division in fission yeast. Mol Biol Cell 26: 256–269. 10.1091/mbc.E14-10-1451 25428987 PMC4294673

[35] Rustici G, Mata J, Kivinen K, Lio P, Penkett CJ, et al (2004) Periodic gene expression program of the fission yeast cell cycle. Nat Genet 36: 809–817. 15195092 10.1038/ng1377

[36] Huang Y, Chew TG, Ge W, Balasubramanian MK (2007) Polarity determinants Tea1p, Tea4p, and Pom1p inhibit division-septum assembly at cell ends in fission yeast. Dev Cell 12: 987–996. 17543869 10.1016/j.devcel.2007.03.015

[37] Liu J, Wang H, Balasubramanian MK (2000) A checkpoint that monitors cytokinesis in *Schizosaccharomyces pombe* . J Cell Sci 113: 1223–1230. 10704373 10.1242/jcs.113.7.1223

[38] Schmidt M, Bowers B, Varma A, Roh DH, Cabib E (2002) In budding yeast, contraction of the actomyosin ring and formation of the primary septum at cytokinesis depend on each other. J Cell Sci 115: 293–302. 11839781 10.1242/jcs.115.2.293

[39] Zhou Z, Munteanu EL, He J, Ursell T, Bathe M, et al (2014) The contractile ring coordinates curvature dependent septum assembly during fission yeast cytokinesis. Mol Biol Cell. 10.1091/mbc.E14-10-1441 PMC4279231 25355954

[40] Sánchez-Díaz A, Marchesi V, Murray S, Jones R, Pereira G, et al (2008) Inn1 couples contraction of the actomyosin ring to membrane ingression during cytokinesis in budding yeast. Nat Cell Biol 10: 395–406. 10.1038/ncb1701 18344988

[41] Nishihama R, Schreiter JH, Onishi M, Vallen EA, Hanna J, et al (2009) Role of Inn1 and its interactions with Hof1 and Cyk3 in promoting cleavage furrow and septum formation in *S*. *cerevisiae* . J Cell Biol 185: 995–1012. 10.1083/jcb.200903125 19528296 PMC2711614

[42] Martín-García R, Valdivieso MH (2006) The fission yeast Chs2 protein interacts with the type-II myosin Myo3p and is required for the integrity of the actomyosin ring. J Cell Sci 119: 2768–2779. 16772338 10.1242/jcs.02998

[43] Pollard LW, Onishi M, Pringle JR, Lord M (2012) Fission yeast Cyk3p is a transglutaminase-like protein that participates in cytokinesis and cell morphogenesis. Mol Biol Cell 23: 2433–2444. 10.1091/mbc.E11-07-0656 22573890 PMC3386208

[44] Bähler J, Wu J-Q, Longtine MS, Shah NG, McKenzie IIIA, et al (1998) Heterologous modules for efficient and versatile PCR-based gene targeting in *Schizosaccharomyces pombe* . Yeast 14: 943–951. 9717240 10.1002/(SICI)1097-0061(199807)14:10<943::AID-YEA292>3.0.CO;2-Y

[45] Shaner NC, Steinbach PA, Tsien RY (2005) A guide to choosing fluorescent proteins. Nat Methods 2: 905–909. 16299475 10.1038/nmeth819

[46] Moreno S, Klar A, Nurse P (1991) Molecular genetic analysis of fission yeast *Schizosaccharomyces pombe* . Methods Enzymol 194: 795–823. 2005825 10.1016/0076-6879(91)94059-l

[47] Kunkel TA (1985) Rapid and efficient site-specific mutagenesis without phenotypic selection. Proc Natl Acad Sci USA 82: 488–492. 3881765 10.1073/pnas.82.2.488 PMC397064

[48] Sambrook J, Russell DW (2001) Molecular cloning: A laboratory manual. Cold Spring Harbor, N.Y.: Cold Spring Harbor Laboratory Press.

[49] Gietz RD, Schiestl RH, Willems AR, Woods RA (1995) Studies on the transformation of intact yeast cells by the LiAc/SS-DNA/PEG procedure. Yeast 11: 355–360. 7785336 10.1002/yea.320110408

[50] Konomi M, Fujimoto K, Toda T, Osumi M (2003) Characterization and behaviour of alpha-glucan synthase in *Schizosaccharomyces pombe* as revealed by electron microscopy. Yeast 20: 427–438. 12673626 10.1002/yea.974

