# Supplementary figures and images for: Cooperation between Paxillin-like Protein Pxl1 and Glucan Synthase Bgs1 Is Essential for Actomyosin Ring Stability and Septum Formation in Fission Yeast
Source: PLoS Genet. 2015 Jul 1;11(7):e1005358. doi: 10.1371/journal.pgen.1005358 (PMC4489101; doi:10.1371/journal.pgen.1005358)

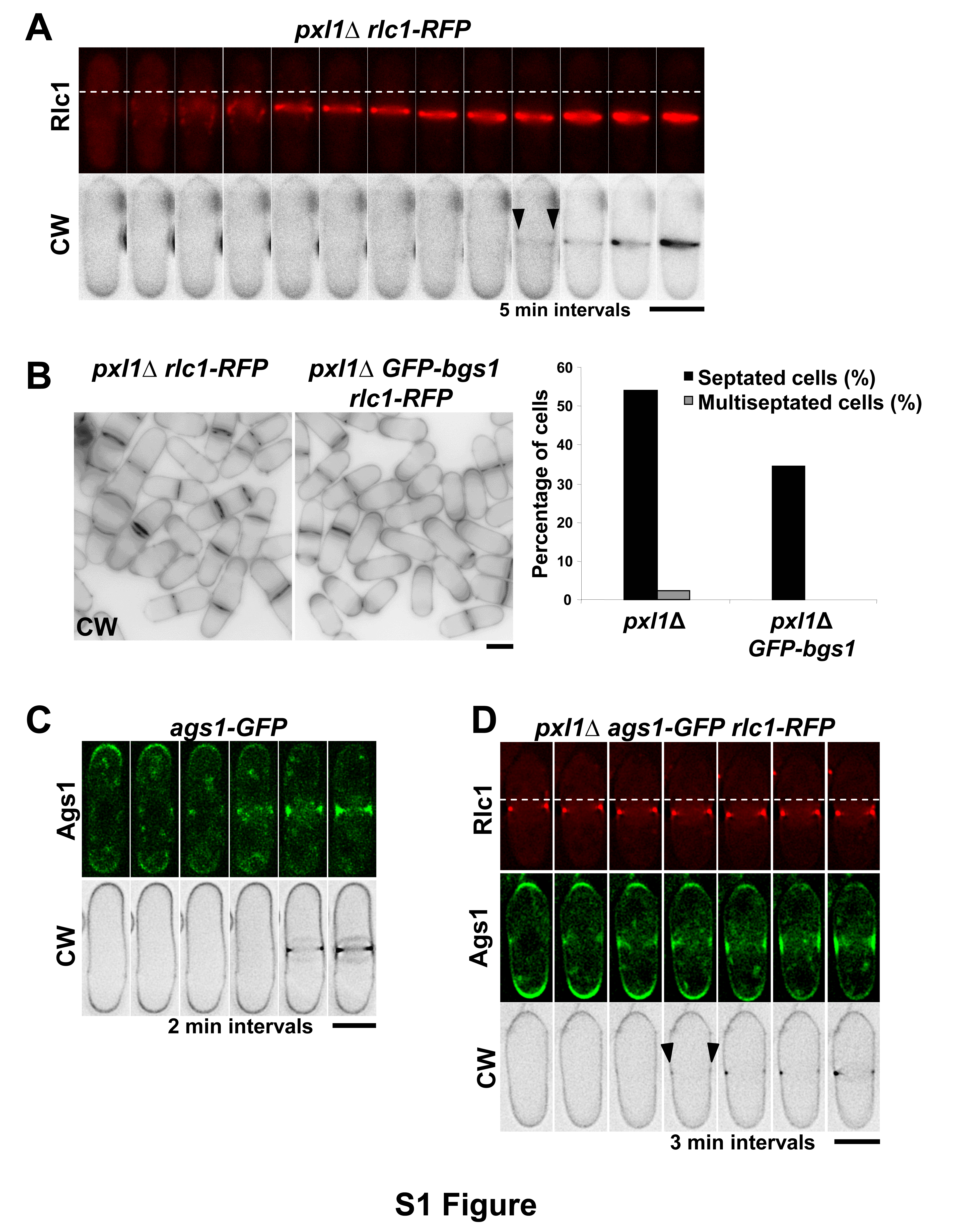

Supplement: S1 Fig — (A) Time series of fluorescence micrographs (one medial z slide, 5 min intervals) of pxl1Δ cells stained with CW and expressing Rlc1-RFP. Arrowheads: septation onset. Dashed line: reference for the ring position in the cell in each micrograph. (B) CW staining images of pxl1Δ cells expressing Rlc1-RFP (left) and GFP-Bgs1 and Rlc1-RFP (right). The graph indicates the percentage of septated (single septum) and multiseptated cells in pxl1Δ rlc1-RFP and pxl1Δ rlc1-RFP GFP-bgs1 + (at least n = 390 cells were quantified for each strain). (C) Time series of fluorescence micrographs (one medial z slide, 2 min intervals) of wild type cells stained with CW and expressing Ags1-GFP. (D) Time series of fluorescence micrographs (one medial z slide, 3 min intervals) of pxl1Δ cells stained with CW, and expressing Ags1-GFP and Rlc1-RFP. Dashed line: reference for the ring position in the cell in each micrograph. Scale bars, 5 μm. (TIF) [file pgen.1005358.s001.tif]

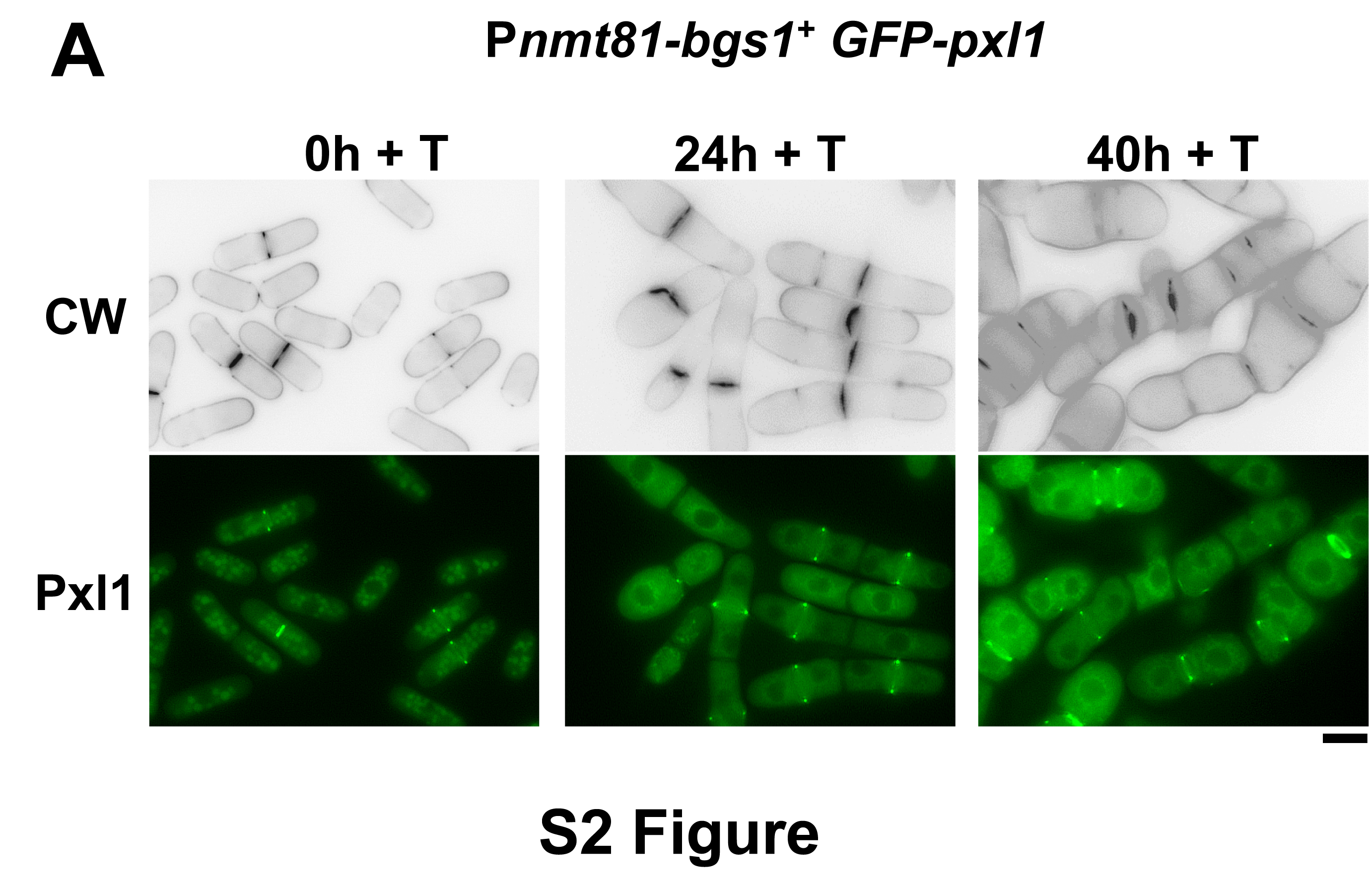

Supplement: S2 Fig — (A) Fluorescence micrographs of Pnmt81-bgs1 + cells stained with CW and expressing GFP-Pxl1. Cells were grown in EMM+S (time 0 h), shifted to EMM+S+T for bgs1 + repression (times 24 and 40 h + T), and imaged at the indicated times. Scale bar, 5 μm. (TIF) [file pgen.1005358.s002.tif]

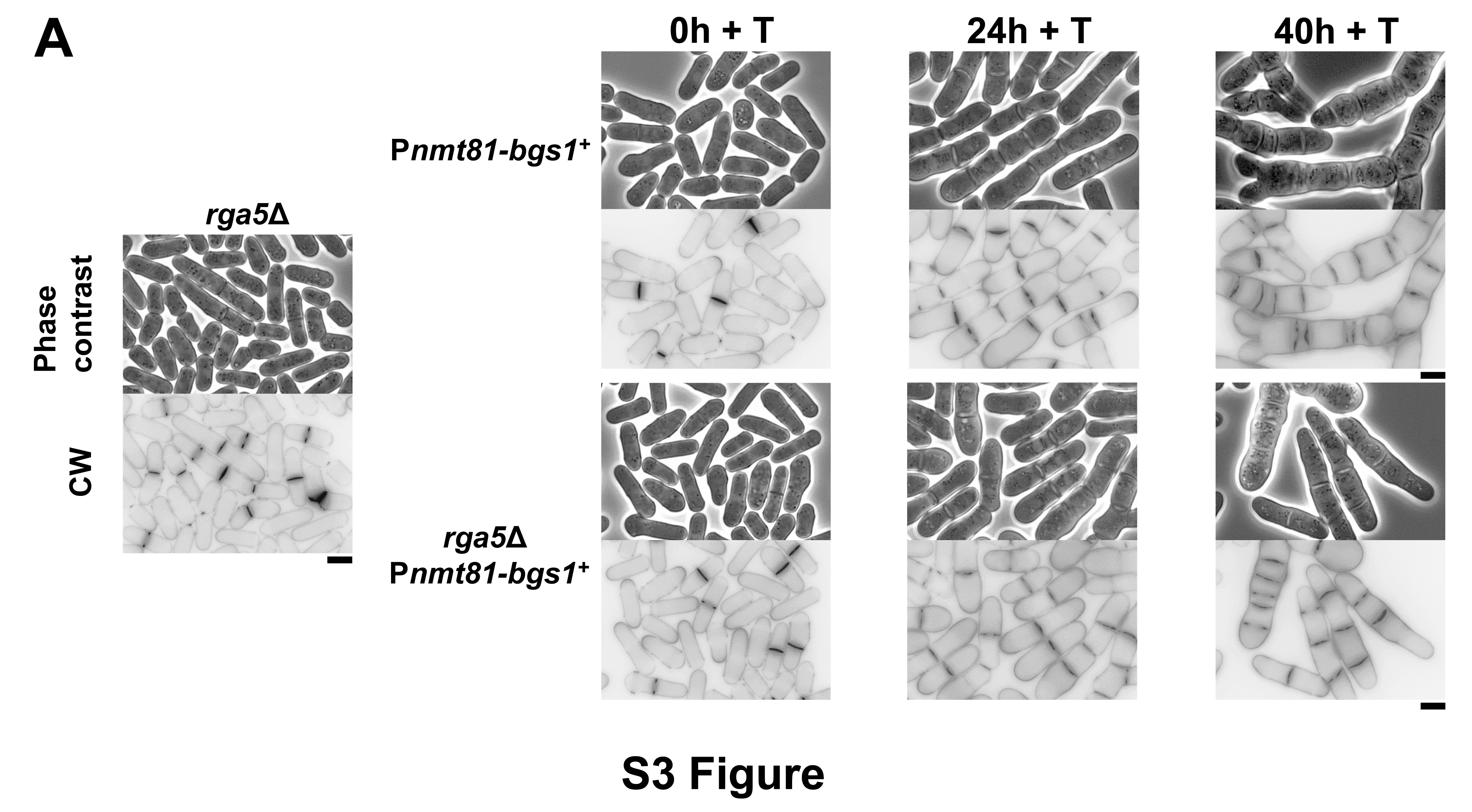

Supplement: S3 Fig — (A) Phase contrast and fluorescence micrographs of rga5Δ, Pnmt81-bgs1 + and rga5Δ Pnmt81-bgs1 + cells stained with CW. Cells were grown in EMM+S (time 0 h), shifted to EMM+S+T for bgs1 + repression (times 24 and 40 h + T), and imaged at the indicated times. Scale bars, 5 μm. (TIF) [file pgen.1005358.s003.tif]

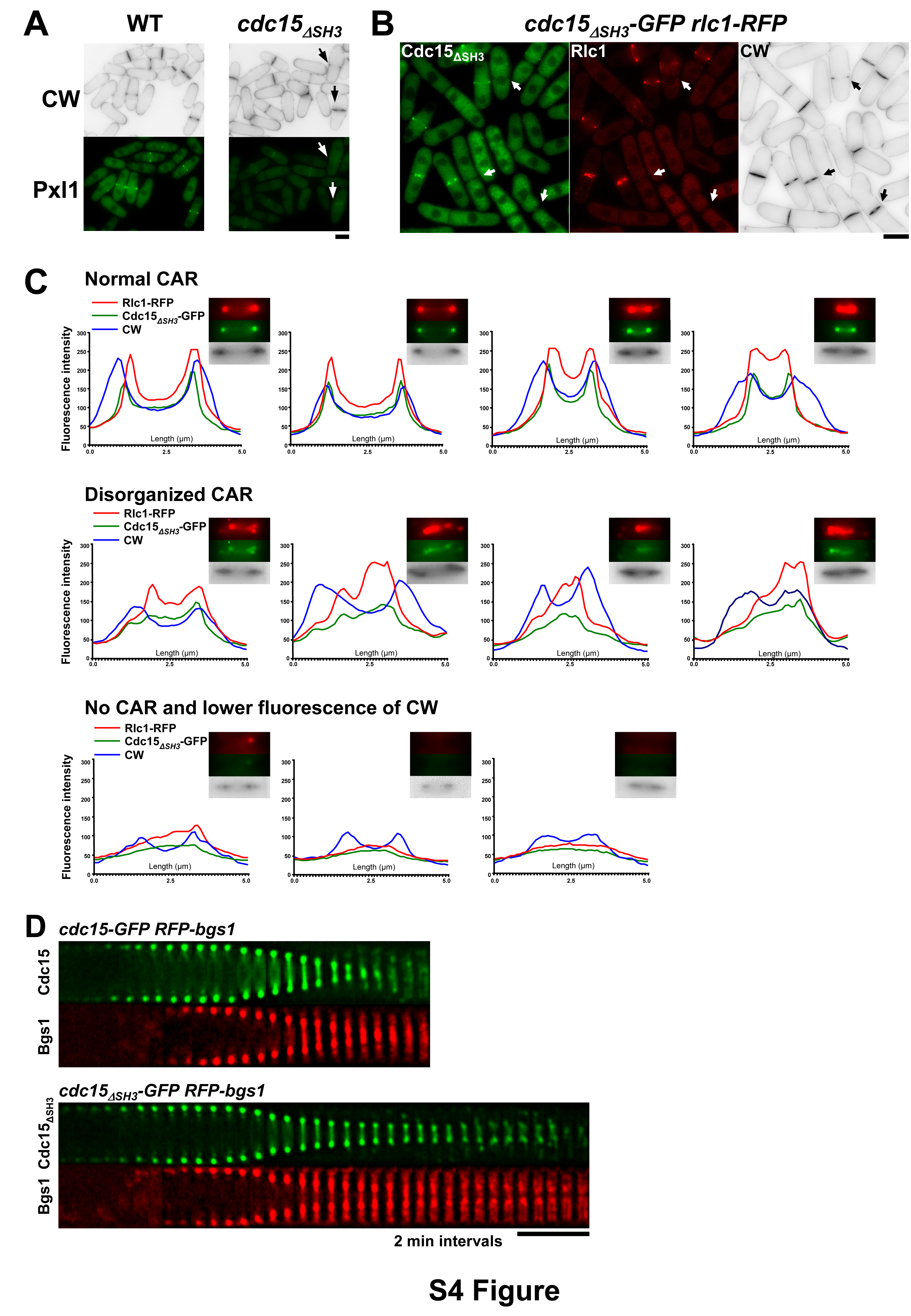

Supplement: S4 Fig — (A) Fluorescence micrographs of wild type and cdc15 ΔSH3 cells stained with CW and expressing GFP-Pxl1. Arrows: Reduction of GFP-Pxl1 fluorescence in open septa. (B) Fluorescence micrographs of cdc15 ΔSH3 cells stained with CW and expressing Cdc15ΔSH3-GFP and Rlc1-RFP. Arrows: Absence of Cdc15 and Rlc1 rings in open septa. (C) Line scans showing the fluorescence intensity of Rlc1-RFP, Cdc15ΔSH3-GFP and CW along open septa in cdc15 ΔSH3 cells. The x-axis represents distance along the septum line and the y-axis is the pixel intensity. Scans were made as described in the Material and Methods section. (D) Kymographs of fluorescence time series (one middle z slide, 2 min intervals) of cdc15-GFP and cdc15 ΔSH3 -GFP cells expressing RFP-Bgs1. Scale bars, 5 μm. (TIF) [file pgen.1005358.s004.tif]

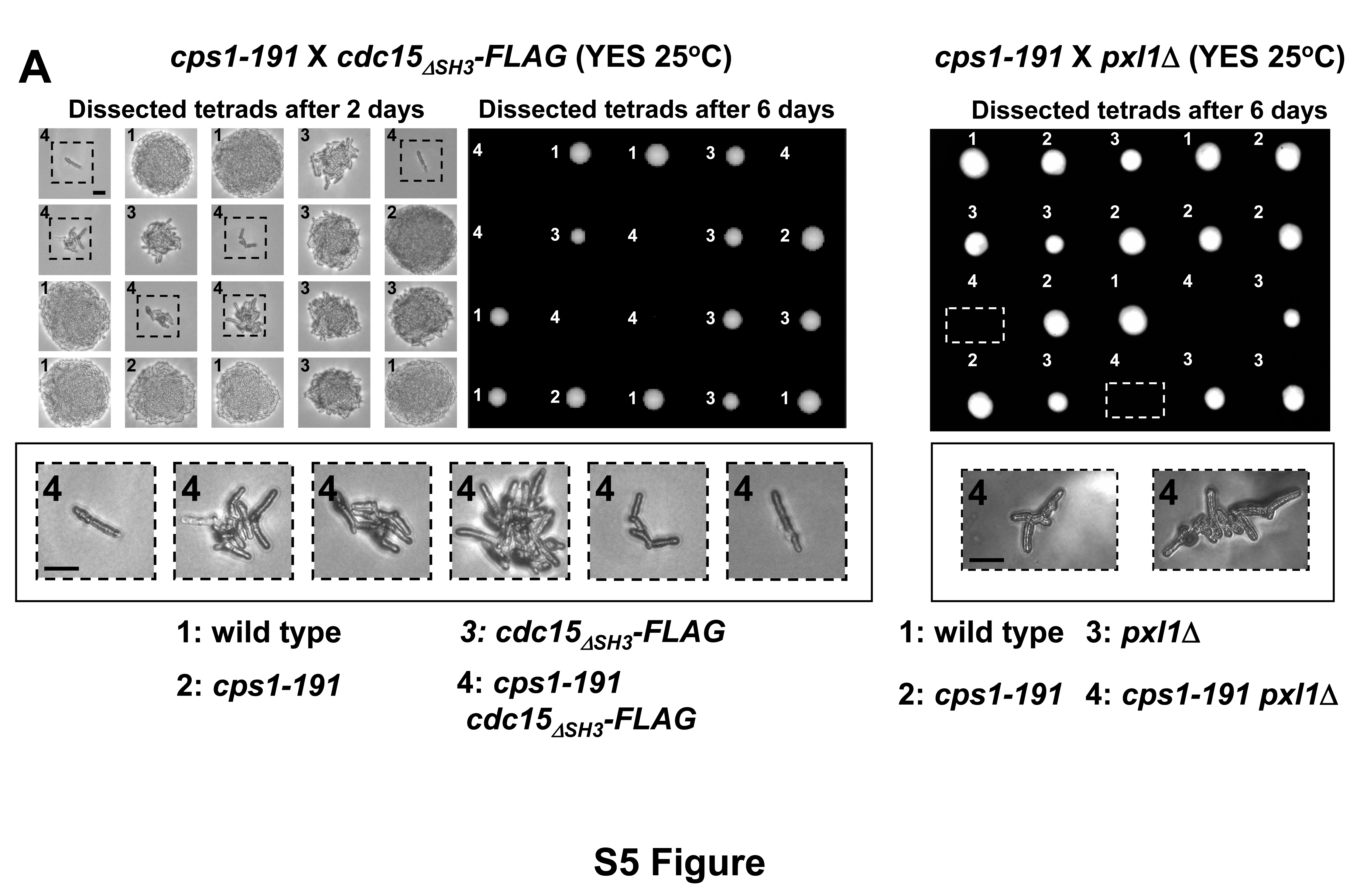

Supplement: S5 Fig — (A) cps1-191 cells were crossed with either cdc15 ΔSH3-FLAG or pxl1Δ cells and tetrads were dissected. Colonies were imaged directly from the plate after 2 (viable colonies) and 6 days (non-viable colonies) of growth in YES plates at 25°C. Broken rectangles indicate non-viable double mutant colonies. Scale bars, 20 μm. (TIF) [file pgen.1005358.s005.tif]
