# Supplementary material for: Cooperation between Paxillin-like Protein Pxl1 and Glucan Synthase Bgs1 Is Essential for Actomyosin Ring Stability and Septum Formation in Fission Yeast
Source: PLoS Genet. 2015 Jul 1;11(7):e1005358. doi: 10.1371/journal.pgen.1005358 (PMC4489101; doi:10.1371/journal.pgen.1005358)
Supplement: S1 Table — (DOC) [file pgen.1005358.s006.doc]

**TABLE S1. Fission yeast strains used in this study.**

Strain Genotype Source

33 972 h^-^ P. Munz^a^

40 *leu1-32* h^+^ Lab collection

419 *leu1-32 ura4-*Δ*18* h^-^ Lab collection

420 *leu1-32 ura4-*Δ*18* h^+^ Lab collection

284 *leu1-32 ura4-*Δ*18 his3-*Δ*1* h^-^ Lab collection

285 *leu1-32 ura4-*Δ*18 his3-*Δ*1* h^+^ Lab collection

1722 *leu1-32 ura4-*Δ*18 his3-*Δ*1 bgs1*Δ*::ura4^+^* P*bgs1^+^::GFP-12A-bgs1^+^:leu1^+^* h^-^ Lab collection

1723 *leu1-32 ura4-*Δ*18 his3-*Δ*1 bgs1*Δ*::ura4^+^* P*bgs1^+^::GFP-12A-bgs1^+^:leu1^+^* h^+^ Lab collection

2438  *leu1-32 ura4-*Δ*18 his3-*Δ*1 bgs4*Δ*::ura4^+^* P*bgs4^+^::GFP-12A-bgs4^+^:leu1^+^* h^+^ Lab collection

3166 *leu1-32 ura4-*Δ*18 his3-*Δ*1 ade6-M210 ags1*Δ 3’UTR*ags1^+^::ags1^+^-12A-GFP-12A:leu1^+^:ura4*^+^ h^-^ Lab collection

3167 *leu1-32 ura4-*Δ*18 his3-*Δ*1 ade6-M210 ags1*Δ 3’UTR*ags1^+^::ags1^+^-12A-GFP-12A:leu1^+^:ura4*^+^ h^+^ Lab collection

1780 *leu1-32 ura4-*Δ*18 his3-*Δ*1 bgs1*Δ*::ura4^+^* P*bgs1^+^::tdTom-12A-bgs1^+^:leu1^+^* h^-^ Lab collection

1813 *leu1-32 ura4-*Δ*18 his3-*Δ*1 bgs1*Δ*::ura4^+^* P*bgs1^+^::tdTom-12A-bgs1^+^:leu1^+^* h^+^ Lab collection

2904  *leu1-32 ura4-*Δ*18 his3-*Δ*1 bgs4*Δ*::ura4^+^* P*bgs4^+^::Cherry-12A-bgs4^+^:leu1^+^* h^-^ Lab collection

4006  *leu1-32 ura4-*Δ*18 ade6-M210 ags1*Δ 3’UTR*ags1^+^::ags1^+^-8A-2XCherry-12A:leu1^+^:ura4*^+^ h^-^ Lab collection

289 *leu1-32 ura4-*Δ*18 cdc15*-*GFP:ura4^+^* h^-^ S. Moreno^b^

983 *leu1-32 ura4-*Δ*18* *cdc15*-*GFP:ura4^+^* h^+^ Lab collection

3265 *ura4-*Δ*18 rlc1^+^-GFP:KanMX6* h^+^ V. Simanis^c^

1755 *leu1-32 ura4-*Δ*18 rlc1^+^-GFP:KanMX6* h^+^ Lab collection

1756 *leu1-32 ura4-*Δ*18 rlc1^+^-GFP:KanMX6* h^-^Lab collection

4868 *leu1-32 ura4-*Δ*18 ade6 rlc1^+^-tdTom:NatMX6* h^-^ Lab collection

4869 *leu1-32 ura4-*Δ*18 ade6 rlc1^+^-tdTom:NatMX6* h^+^ Lab collection

2525 *leu1-32 ura4-*Δ*18 leu1::GFP-atb2^+^:ura4^+^* h^+^ V. Simanis^c^

4797 *leu1-32 ura4-*Δ*18 his3D-1 leu1::GFP-atb2^+^:ura4^+^* h^+^ This study

5043 *leu1-32 ura4-*Δ*18 Cherry-atb2^+^:Aur1* h^+^ T. Toda^d^

2048 *leu1-32 ura4-*Δ*18 his3-*Δ*1* P*bgs1^+^*Δ::P*nmt1-*81X-*bgs1*^+^*:ura4^+^* h^-^ Lab collection

1483 *leu1-32 ura4-*Δ*18 his3-*Δ*1 ade6-M210 Pbgs1^+^*Δ::P*nmt1-*81X-*bgs1*^+^*:ura4^+^* h^-^ Lab collection

3537 *leu1-32 ura4-*Δ*18 pxl1*Δ*::KanMX6* h^+^ Lab collection

3538 *leu1-32 ura4-*Δ*18 pxl1*Δ*::KanMX6* *GFP-pxl1^+^:leu1^+^* h^+^ Lab collection

4873 *leu1-32 ura4-*Δ*18 pxl1*Δ*::ura4^+^* *rlc1^+^-tdTom:NatMX6* h^-^ Lab collection

5006 *leu1-32 ura4-*Δ*18 his3-Δ1 leu1::GFP-atb2^+^:ura4^+^* *rlc1^+^-tdTom:NatMX6* h^+^ This study

4992 *leu1-32 ura4-*Δ*18 pxl1*Δ*::ura4^+^* *leu1:GFP-atb2^+^::ura4^+^* *rlc1^+^-tdTom:NatMX6* h^-^ This study

4973 *leu1-32 ura4-*Δ*18 ade6-M216 ags1*Δ *pxl1*Δ*::ura4^+^* *rlc1^+^-tdTom:NatMX6*

3’UTR*ags1^+^::ags1^+^-12A-GFP-12A:leu1^+^:ura4*^+^ h^+^  This study

4975 *leu1-32 ura4-*Δ*18 bgs1*Δ*::ura4^+^ pxl1*Δ*::ura4^+^* *rlc1^+^-tdTom:NatMX6* P*bgs1^+^::GFP-12A-bgs1^+^:leu1^+^* h^+^ This study

5590 *leu1-32 ura4-*Δ*18 his3-Δ1 ade6-M210 ags1*Δ *leu1::GFP-atb2^+^:ura4^+^* *rlc1^+^-tdTom:NatMX6*

3’UTR*ags1^+^::ags1^+^-12A-GFP-12A:leu1^+^:ura4*^+^ h^+^ This study

5591 *leu1-32 ura4-*Δ*18 his3-Δ1 bgs1*Δ*::ura4^+^ leu1::GFP-atb2^+^:ura4^+^ rlc1^+^-tdTom:NatMX6*

P*bgs1^+^::GFP-12A-bgs1^+^:leu1^+^* h^+^ This study

5579 *leu1-32 ura4-*Δ*18 ade6-M210 ags1*Δ *pxl1*Δ*::ura4^+^ leu1::GFP-atb2^+^:ura4^+^*

*rlc1^+^-tdTom:NatMX6* 3’UTR*ags1^+^::ags1^+^-12A-GFP-12A:leu1^+^:ura4*^+^ h^+^ This study

5581 *leu1-32 ura4-*Δ*18 bgs1*Δ*::ura4^+^pxl1*Δ*::ura4^+^ leu1::GFP-atb2^+^:ura4^+^*

*rlc1^+^-tdTom:NatMX6* P*bgs1^+^::GFP-12A-bgs1^+^:leu1^+^* h^+^ This study

Strain Genotype Source

972 *cps1-191 ura4-*Δ*18* h^-^ M. Balasubramanian^e^

973 *cps1-191 ura4-*Δ*18 ade6-M210 lys1-131* h^-^ M. Balasubramanian^e^

1024 *cps1-191 leu1-32 ura4-*Δ*18* h^-^ Lab collection

1025 *cps1-191 leu1-32 ura4-*Δ*18* h^+^ Lab collection

5021 *cps1-191 leu1-32 ura4-*Δ*18 rlc1^+^-GFP:KanMX6* h^+^ This study

5032 *cps1-191¿leu1-32? ura4-*Δ*18 rlc1^+^-tdTom:NatMX6* h^-^ This study

5016 *leu1-32 GFP-psy1^+^:leu1^+^* h^90^ C. Shimoda^f^

5045 *leu1-32 ura4-*Δ*18 rlc1^+^-tdTom:NatMX6 GFP-psy1^+^:leu1^+^* h^+^ Lab collection

5064 *cps1-191 leu1-32 ura4-*Δ*18 rlc1^+^-tdTom:NatMX6 GFP-psy1^+^:leu1^+^* h^+^ This study

5083 *leu1-32 ura4-*Δ*18 bgs4*Δ*::ura4^+^* *rlc1^+^-tdTom:NatMX6* P*bgs4^+^::GFP-12A-bgs4^+^:leu1^+^* h^+^ This study

5084 *leu1-32 ura4-*Δ*18 ade6-M216 ags1*Δ *rlc1^+^-tdTom:NatMX6*

3’UTR*ags1^+^::ags1^+^-12A-GFP-12A:leu1^+^:ura4*^+^ h^+^ This study

5085 *cps1-191 ¿leu1-32? ura4-*Δ*18 bgs4*Δ*::ura4^+^* *rlc1^+^-tdTom:NatMX6* P*bgs4^+^::GFP-bgs4^+^:leu1^+^* h^+^ This study

5087 *cps1-191* *¿leu1-32? ura4-*Δ*18 ade6-M216 ags1*Δ *rlc1^+^-tdTom:NatMX6*

3’UTR*ags1^+^::ags1^+^-12A-GFP-12A:leu1^+^:ura4*^+^ h^+^ This study

4967 *leu1-32 ura4-*Δ*18* P*nmt1-*41X-*pxl1*^+^*:KanMX6* *rlc1^+^-tdTom:NatMX6* h^+^ Lab collection

5031 *cps1-191 ura4-*Δ*18* P*nmt1-*41X-*pxl1*^+^*:KanMX6* *rlc1^+^-tdTom:NatMX6* h^-^ This study

3920 *leu1-32 ura4-*Δ*18 pxl1*Δ*::KanMX6* *rlc1^+^-GFP:KanMX6* h^+^ This study

3778 *leu1-32 ura4-*Δ*18 ade6 pxl1*Δ*::KanMX6* P*bgs1^+^*Δ::P*nmt1-*81X-*bgs1*^+^*:ura4^+^* h^-^ This study

3839 *ura4-*Δ*18 ade6-M210 rlc1^+^-GFP:KanMX6* P*bgs1^+^*Δ::P*nmt1-*81X-*bgs1*^+^*:ura4^+^* h^-^ This study

3841 *ura4-*Δ*18 ade6-M210 pxl1*Δ*::KanMX6* *rlc1^+^-GFP:KanMX6* P*bgs1^+^*Δ::P*nmt1-*81X-*bgs1*^+^*:ura4^+^* h^+^ This study

3683 *leu1-32 ura4-*Δ*18 pxl1*Δ*::KanMX6* *GFP-pxl1^+^:leu1^+^* P*bgs1^+^*Δ::P*nmt1-*81X-*bgs1*^+^*:ura4^+^* h^+^ This study

2170  *leu1-32 ura4-*Δ*18 his3-*Δ*1 bgs4*Δ*::ura4^+^* *rlc1^+^-tdTom:NatMX6* P*bgs4^+^::GFP-12A-bgs4^+^:leu1^+^* h^+^ This study

2174 *leu1-32 ura4-*Δ*18 his3-*Δ*1 ade6-M216 ags1*Δ *rlc1^+^-tdTom:NatMX6*

3’UTR*ags1^+^::ags1^+^-12A-GFP-12A:leu1^+^:ura4*^+^ h^+^ This study

5604 *leu1-32 ura4-*Δ*18 ade6-M216 ags1*Δ *rlc1^+^-tdTom:NatMX6*

P*bgs1^+^*Δ::P*nmt1-*81X-*bgs1*^+^*:ura4^+^* 3’UTR*ags1^+^::ags1^+^-12A-GFP-12A:leu1^+^:ura4*^+^ h^-^ This study

5599 *leu1-32 ura4-*Δ*18 ade6-M216 ags1*Δ *pxl1*Δ*::KanMX6* *rlc1^+^-tdTom:NatMX6*

P*bgs1^+^*Δ::P*nmt1-*81X-*bgs1*^+^*:ura4^+^* 3’UTR*ags1^+^::ags1^+^-12A-GFP-12A:leu1^+^:ura4*^+^ h^-^ This study

5605 *leu1-32 ura4-*Δ*18 ade6-M210 bgs4*Δ*::ura4^+^* *rlc1^+^-tdTom:NatMX6*

P*bgs1^+^*Δ::P*nmt1-*81X-*bgs1*^+^*:ura4^+^* P*bgs4^+^::GFP-12A-bgs4^+^:leu1^+^* h^+^ This study

5600 *leu1-32 ura4-*Δ*18 bgs4*Δ*::ura4^+^* *pxl1*Δ*::KanMX6* *rlc1^+^-tdTom:NatMX6*

P*bgs1^+^*Δ::P*nmt1-*81X-*bgs1*^+^*:ura4^+^* P*bgs4^+^::GFP-12A-bgs4^+^:leu1^+^* h^-^ This study

5247 *leu1-32 ura4-*Δ*18 ade6-M216 rga5*Δ*::KanMX6* h^+^ Lab collection

5273 *leu1-32 ura4-*Δ*18 ade6-M216 rga5*Δ*::KanMX6* P*bgs1^+^*Δ::P*nmt1-*81X-*bgs1*^+^*:ura4^+^* h^-^ This study

5249 *leu1-32 ura4-*Δ*18 pxl1*Δ*::ura4^+^* *GFP-pxl1^+^:leu1^+^* h^-^ Lab collection

5117 *leu1-32 ura4-*Δ*18 ade6 cdc15*Δ*SH3-FLAG:KanMX6* h^+^ K. Gould^g^

5118 *leu1-32 ura4-*Δ*18 ade6 cdc15*Δ*SH3-FLAG:KanMX6* h^-^ K. Gould^g^

5174 *leu1-32 ura4-*Δ*18 pxl1*Δ*::KanMX6* *GFP-pxl1^+^:leu1^+^ cdc15*Δ*SH3-FLAG:KanMX6* h^+^ This study

5596 *leu1-32 ura4-*Δ*18 Cherry-atb2^+^:Aur1 cdc15*Δ*SH3-FLAG:KanMX6* h^+^ This study

5597 *leu1-32 ura4-*Δ*18 pxl1*Δ*::ura4^+^* *GFP-pxl1^+^:leu1^+^ Cherry-atb2^+^:Aur1* h^-^ This study

5598 *leu1-32 ura4-*Δ*18 cdc15*Δ*SH3-FLAG:KanMX6 pxl1*Δ*:: ura4^+^*

*GFP-pxl1^+^:leu1^+^ Cherry-atb2^+^:Aur1* h^-^ This study

5606 *leu1-32 ura4-*Δ*18 bgs1*Δ*::ura4^+^* *Cherry-atb2^+^:Aur1* P*bgs1^+^::GFP-12A-bgs1^+^:leu1^+^* h^-^ This study

5607 *leu1-32 ura4-*Δ*18 bgs1*Δ*::ura4^+^* *cdc15*Δ*SH3-FLAG:KanMX6 Cherry-atb2^+^:Aur1*

P*bgs1^+^::GFP-12A-bgs1^+^:leu1^+^* h^+^ This study

5609 *leu1-32 ura4-*Δ*18 bgs1*Δ*::ura4^+^* *pxl1*Δ*::ura4^+^* *Cherry-atb2^+^:Aur1* P*bgs1^+^::GFP-12A-bgs1^+^:leu1^+^* h^-^ This study

Strain Genotype Source

4888 *leu1-32 ura4-*Δ*18 ade6 cdc15*Δ*SH3-GFP:KanMX6* h^+^ K. Gould^g^

5039 *leu1-32 ura4-*Δ*18 cdc15*Δ*SH3-GFP:KanMX6* P*bgs1^+^*Δ::P*nmt1-*81X-*bgs1*^+^*:ura4^+^* h^+^ This study

5574 *leu1-32 ura4-*Δ*18 bgs4*Δ*::ura4^+^* P*bgs1^+^*Δ::P*nmt1-*81X-*bgs1*^+^*:ura4^+^* P*bgs4^+^::Cherry-12A-bgs4^+^:leu1^+^* h^+^ This study

5571 *leu1-32 ura4-*Δ*18 bgs4*Δ*::ura4^+^* *cdc15*Δ*SH3-GFP:KanMX6*

P*bgs1^+^*Δ::P*nmt1-*81X-*bgs1*^+^*:ura4^+^* P*bgs4^+^::Cherry-12A-bgs4^+^:leu1^+^* h^+^ This study

5578 *leu1-32 ura4-*Δ*18 ade6-M210* *ags1*Δ P*bgs1^+^*Δ::P*nmt1-*81X-*bgs1*^+^*:ura4^+^*

3’UTR*ags1^+^::ags1^+^-8A-2XCherry-12A:leu1^+^:ura4*^+^ h^+^ This study

5575 *leu1-32 ura4-*Δ*18 ade6-M210* *ags1*Δ *cdc15*Δ*SH3-GFP:KanMX6*

P*bgs1^+^*Δ::P*nmt1-*81X-*bgs1*^+^*:ura4^+^* 3’UTR*ags1^+^::ags1^+^-8A-2XCherry-12A:leu1^+^:ura4*^+^ h^+^ This study

5186 *leu1-32 ura4-*Δ*18* his3-Δ1 *GFP-12A-cps1-191:leu1^+^* P*bgs1^+^*Δ::P*nmt1-*81X-*bgs1*^+^*:ura4^+^* h^-^ This study

5349 *leu1-32 ura4-*Δ*18 pxl1*Δ*::KanMX6 GFP-12A-cps1-191:leu1^+^* P*bgs1^+^*Δ::P*nmt1-*81X-*bgs1*^+^*:ura4^+^* h^-^ This study

5352 *leu1-32 ura4-*Δ*18 ade6-M216 cdc15*Δ*SH3-HA:KanMX6 GFP-12A-cps1-191:leu1^+^*

P*bgs1^+^*Δ::P*nmt1-*81X-*bgs1*^+^*:ura4^+^* h^-^ This study

5079 *leu1-32 ura4-*Δ*18* P*nmt1-*81X-*pxl1*^+^*:KanMX6* h^-^ Lab collection

4998 *leu1-32 ura4-*Δ*18 cdc15*-*GFP:ura4^+^* P*nmt1-*81X-*pxl1*^+^*:KanMX6* h^+^ Lab collection

3738 *leu1-32 ura4-*Δ*18 ade6-M210* *ags1*Δ P*nmt1-*81X-*pxl1*^+^*:KanMX6*

3’UTR*ags1^+^::ags1^+^-8A-2XCherry-12A:leu1^+^:ura4*^+^ h^+^ This study

2483 *leu1-32 ura4-*Δ*18 ade6-M210* *ags1*Δ *cdc15*-*GFP:ura4^+^* P*nmt1-*81X-*pxl1*^+^*:KanMX6*

3’UTR*ags1^+^::ags1^+^-8A-2XCherry-12A:leu1^+^:ura4*^+^ h^+^ This study

5260 *leu1-32 ura4-*Δ*18 bgs4*Δ*::ura4^+^* P*nmt1-*81X-*pxl1*^+^*:KanMX6* P*bgs4^+^::Cherry-12A-bgs4^+^:leu1^+^* h^+^ This study

3940 *leu1-32 ura4-*Δ*18 bgs4*Δ*::ura4^+^* *cdc15*-*GFP:ura4^+^* P*nmt1-*81X-*pxl1*^+^*:KanMX6*

P*bgs4^+^::Cherry-12A-bgs4^+^:leu1^+^* h^+^ This study

5010 *leu1-32 ura4-*Δ*18 cdc15*Δ*SH3-GFP:KanMX6 rlc1^+^-tdTom:NatMX6* h^+^ This study

5091 *leu1-32 ura4-*Δ*18 his3-*Δ*1* *ade6-M210 bgs1*Δ*::ura4^+^* *cdc15*Δ*SH3-GFP:KanMX6*

P*bgs1^+^::tdTom-12A-bgs1^+^:leu1^+^* h^-^ This study

5145 *leu1-32 ura4-*Δ*18 bgs1*Δ*::ura4^+^* *cdc15*-*GFP:ura4^+^* P*bgs1^+^::tdTom-12A-bgs1^+^:leu1^+^* h^+^ This study

^a^ Institute of General Microbiology, University of Bern, Switzerland.

^b^ Instituto de Biología Funcional y Genómica (Consejo Superior de Investigaciones Científicas / Universidad de Salamanca), Salamanca, Spain.

^c^ School of Life Sciences, École Polytechnique Fédérale de Lausanne (EPFL), Lausanne, Switzerland.

^d^ Laboratory of Cell Regulation, Cancer Research UK, London Research Institute, 44 Lincoln's Inn Fields, London WC2A 3LY, UK.

^e^ Warwick Medical School, The University of Warwick, Coventry, CV4 7AL, UK.

^f^ Department of Biology, Graduate School of Science, Osaka City University, Osaka, Japan.

^g^ Department of Cell and Developmental Biology, Vanderbilt University School of Medicine, Nashville, Tennessee 37232, USA.
